# Aspergillus fumigatus In-Host HOG Pathway Mutation for Cystic Fibrosis Lung Microenvironment Persistence

**DOI:** 10.1128/mBio.02153-21

**Published:** 2021-08-31

**Authors:** Brandon S. Ross, Lotus A. Lofgren, Alix Ashare, Jason E. Stajich, Robert A. Cramer

**Affiliations:** a Department of Microbiology and Immunology, Geisel School of Medicine at Dartmouth, Hanover, New Hampshire, USA; b Department of Microbiology and Plant Pathology, Institute for Integrative Genome Biology, University of California Riverside, Riverside, California, USA; c Department of Medicine, Dartmouth-Hitchcock Medical Center, Lebanon, New Hampshire, USA; Duke University Medical Center

**Keywords:** *Aspergillus fumigatus*, cystic fibrosis, chronic infection, hypoxia, osmotic stress, oxidative stress, genomics, MAP kinases, pathogenesis

## Abstract

The prevalence of Aspergillus fumigatus colonization in individuals with cystic fibrosis (CF) and subsequent fungal persistence in the lung is increasingly recognized. However, there is no consensus for clinical management of A. fumigatus in CF individuals, due largely to uncertainty surrounding A. fumigatus CF pathogenesis and virulence mechanisms. To address this gap in knowledge, a longitudinal series of A. fumigatus isolates from an individual with CF were collected over 4.5 years. Isolate genotypes were defined with whole-genome sequencing that revealed both transitory and persistent A. fumigatus in the lung. Persistent lineage isolates grew most readily in a low-oxygen culture environment, and conidia were more sensitive to oxidative stress-inducing conditions than those from nonpersistent isolates. Closely related persistent isolates harbored a unique allele of the high-osmolarity glycerol (HOG) pathway mitogen-activated protein kinase kinase, Pbs2 (*pbs2^C2^*). Data suggest this novel *pbs2^C2^* allele arose *in vivo* and is necessary for the fungal response to osmotic stress in a low-oxygen environment through hyperactivation of the HOG (SakA) signaling pathway. Hyperactivation of the HOG pathway through *pbs2^C2^* comes at the cost of decreased conidial stress resistance in the presence of atmospheric oxygen levels. These novel findings shed light on pathoadaptive mechanisms of A. fumigatus in CF, lay the foundation for identifying persistent A. fumigatus isolates that may require antifungal therapy, and highlight considerations for successful culture of persistent Aspergillus CF isolates.

## INTRODUCTION

The prevalence of fungal colonization in cystic fibrosis (CF) individuals’ lungs has become increasingly recognized (1–3). CF arises from defects in the human cystic fibrosis transmembrane conductance regulator (hCFTR) that result in a thickened mucus layer over lung epithelial cells (4). The dense layer of mucins, oligosaccharides, and extracellular DNA blocks normal mucociliary clearance, an important innate defense against environmental debris and inhaled microbes (5). A reduction in mucociliary clearance in combination with mucus’ nutrient content renders CF airways susceptible to both transient microbial colonization and persistent infection (6). Although decidedly different in duration and evolutionary potential, both of these host-microbe interactions lead to the development of recurrent inflammation, steep oxygen and osmolarity gradients, and progressive fibrotic tissue damage resulting in lung function decline (7–10).

It is well documented that several fungal genera that cause severe infections in immunocompromised patient populations are also commonly isolated from CF patient airways (11–13). Aspergillus fumigatus is the most commonly isolated filamentous fungal species from CF patients (1, 14). Estimates point to A. fumigatus colonization being present in up to ∼60% of CF patients, and recent data demonstrate that initial colonization occurs as early as infancy (15, 16). Aspergillus-specific diseases affecting CF patients include Aspergillus bronchitis (AB) and allergic bronchopulmonary aspergillosis (ABPA), each of which are associated with persistent fungal colonization and worse patient outcomes (17). Despite evidence that A. fumigatus persistence can last for years in CF patients and reports that persistent fungal colonization and associated AB and ABPA negatively impact patient outcomes, there is little consensus for the importance of direct clinical management of Aspergillus culture positivity in CF patients (18–20). This is in contrast to bacterial persistence, where dynamic and persistent bacterial populations are widely understood to contribute to worsening outcomes in CF (21, 22). Consequently, patients with CF are routinely treated with antibiotics but far less frequently with antifungals, even in the face of persistent fungal colonization (23). A better understanding of the pathogenesis and virulence mechanisms of A. fumigatus in patients with CF is expected to help identify in what context antifungal therapies should be considered.

Analyses of bacterial isolate series from patients with CF have been key to understanding bacterial pathogenesis and virulence in CF disease progression. For example, genomic and genetic analyses of CF bacterial isolates have revealed significant intraspecies diversity, phenotypic convergence, and in-host evolution in microbes such as Pseudomonas aeruginosa and *Burkholderia* species, among others (24, 25). While intraspecies diversity in A. fumigatus is associated with a spectrum of disease manifestations, there is little consensus as to whether A. fumigatus culture positivity in CF patients is a sign of infection or transient colonization (18, 26). Until recently, efforts to examine longitudinal genotypic diversity in CF A. fumigatus isolates have relied on lower-resolution genotyping techniques in contrast to bacterial whole-genome sequence analyses (27–29). Moreover, phenotypic analysis of A. fumigatus CF isolates has largely focused on antifungal drug susceptibility and resistance (30). A recent study from Engel and colleagues reported parasexual recombination as a potential mechanism for persistence of A. fumigatus in chronic infections, but the implications of this genetic diversity for phenotypes outside antifungal resistance are unclear (31). There remains a lack of longitudinal studies that directly address the genotypic and CF-relevant phenotypic characteristics of persistent A. fumigatus isolates. Longitudinal studies have significant potential to shed light on CF-specific A. fumigatus pathogenesis mechanisms similarly to studies of CF-associated bacteria (32).

Here, we defined the genotypes and phenotypes of a longitudinal series of A. fumigatus isolates collected from a patient with CF over the course of ∼4.5 years (the AF100 series). Whole-genome sequencing and phylogenetic analyses of 29 isolates from 12 different time points revealed extensive genetic diversity and two lineages of persistent isolates. One lineage of 5 persistent isolates has evidence of positive natural selection. Isolates from this persistent lineage have conidia with a striking increased sensitivity to growth under ambient oxygen conditions (normoxia) and oxidative stress-inducing culture conditions but also an increase in osmotic stress resistance compared to that of nonpersistent and reference isolates. Conidial phenotypes result in part from a unique allele of *pbs2*, the mitogen-activated protein kinase (MAPK) kinase associated with the high-osmolarity glycerol (HOG) MAPK pathway that most likely arose *in vivo* during growth in the CF airway (33, 34). Furthermore, we observed that this unique *pbs2* allele promotes hyphal osmotic and oxidative stress resistance at the cost of conidial oxidative stress susceptibility. Taken together, these results reveal new insights into pathoadaptive mechanisms of persistent A. fumigatus CF isolates and provide support for the importance of further genetic and phenotypic studies of A. fumigatus CF isolates in the context of more patients and disease progression outcomes.

## RESULTS

### The AF100 series is a longitudinal series of Aspergillus fumigatus isolates from a single patient with CF.

**FIG 1:**
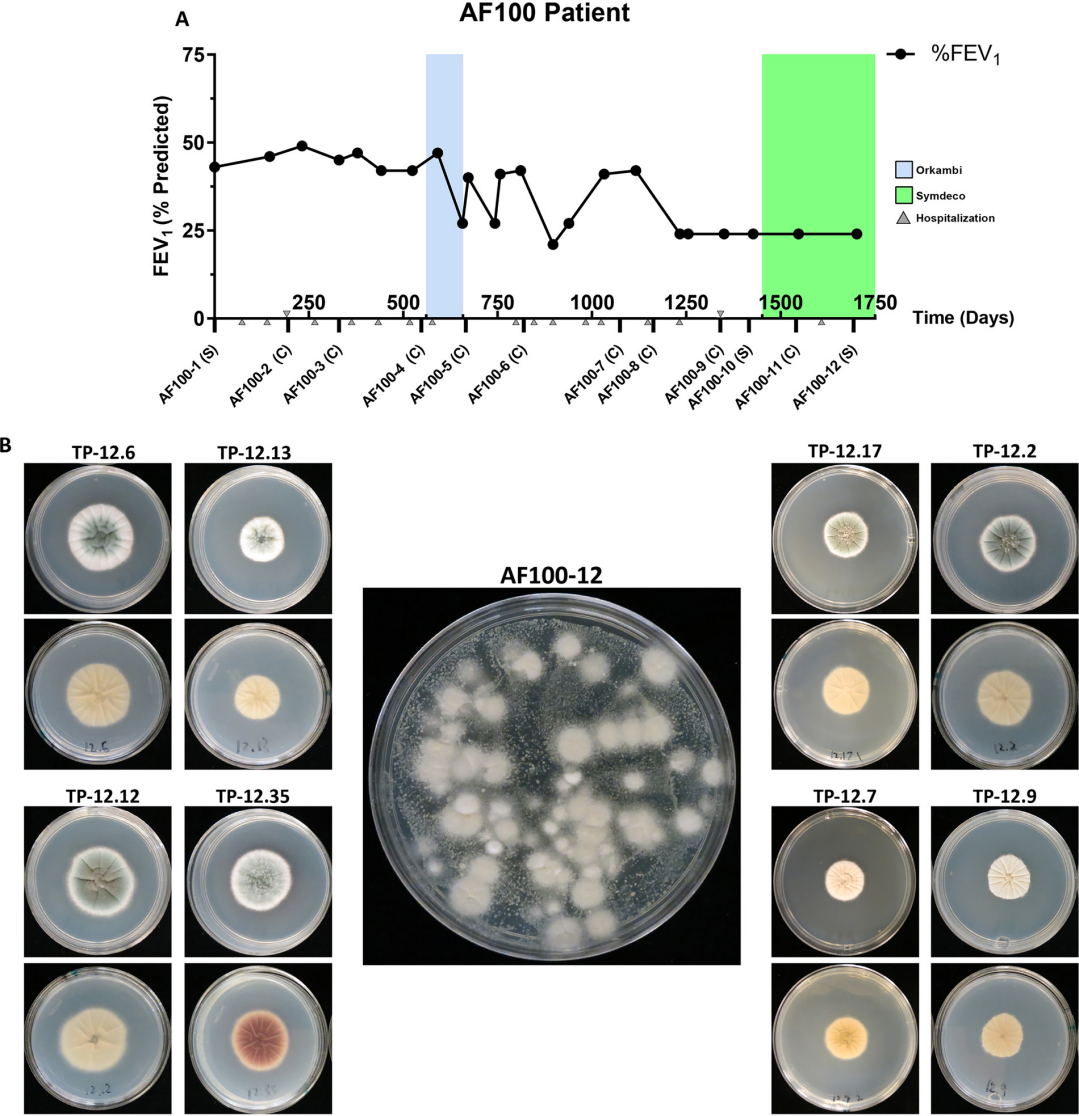
The longitudinal Aspergillus fumigatus isolate series from the AF100 cystic fibrosis individual reveals morphological diversity. (A) Timeline of sample isolation, forced expiratory volume in 1 s (FEV_1_), and cystic fibrosis medication history for the AF100 individual over the time covered in this study. The letter below each AF100 sample represents the sample type: C, individual/subcultured colony sample from clinical microbiology laboratory at Dartmouth Hitchcock Medical Center; S, sputum sample. (B) Single colony images highlighting morphology of front (top image) and back (bottom image) of Aspergillus minimal medium (AMM) plates for representative colonies picked from plated AF100-12 sputum (center). Sputum was initially cultured on Sabouraud dextrose agar for 30 h under normoxia, and single cultures were subsequently cultured on AMM for 48 h at 37°C under normoxia.

We investigated a series of A. fumigatus isolates collected from a person living with an F508del/F508del CFTR genotype, herein called the AF100 series. The AF100 series is an ongoing longitudinal collection of >30 isolates collected from patient sputum over an ∼4.5-year period (Fig. 1A). Over the course of the study, the patient exhibited a steady decrease in forced expiratory volume in 1 s (%FEV_1_), a clinical metric used to define overall lung capacity and function. The patient also had multiple hospitalizations associated with persistent A. fumigatus culture positivity but was not treated with antifungal therapy. The patient began showing consistent A. fumigatus culture positivity starting in the year 2005. Sputum samples for each of the 12 time points are designated “AF100-X,” where X corresponds to the time point in the series. Fungal isolates were obtained from the AF100 sputum samples in one of two ways: as individual colonies subcultured from primary sputum cultures by staff at the Clinical Microbiology Laboratory at Dartmouth-Hitchcock Medical Center (DHMC) and as a collection of multiple different colonies picked from patient sputum samples cultured in the Cramer laboratory (labeled C and S, respectively, in Fig. 1A). Isolates from the AF100 samples were subsequently designated “TP-X,” so that TP-2 is the isolate from the AF100-2 sample, and TP-3 is from AF100-3, etc. It is important to note sputum samples that allowed us to pick multiple isolates (AF100-1, AF100-10, AF100-11, and AF100-12) required further numbering to differentiate isolates (i.e., TP-12.7, TP-12.9, TP-12.13, etc.).

We were initially curious to understand the level of A. fumigatus diversity in an individual sputum sample; to do this, we used a preliminary qualitative screen for morphological diversity in the AF100-12 sputum sample. After culturing the sputum on Sabouraud’s dextrose agar medium for 30 h and subculturing individual colonies to Aspergillus minimal medium (AMM) for 48 h, we observed a high degree of morphological diversity (Fig. 1B). We recovered at least 8 unique colony morphotypes from the AF100-12 sputum sample, with striking variation in coloration, furrowing, aerial hyphae, and colony diameter (Fig. 1B). These observations are consistent with previous reports on the presence of multiple phenotypically diverse A. fumigatus isolates in the CF lung at any given time (28, 35, 36). To better understand these observations and determine potential genetic relationships among the AF100 isolates, we turned to whole-genome sequencing.

### Whole-genome sequencing and phylogenetic analyses reveal significant genetic diversity and 2 persistent lineages within the AF100 series.

**FIG 2:**
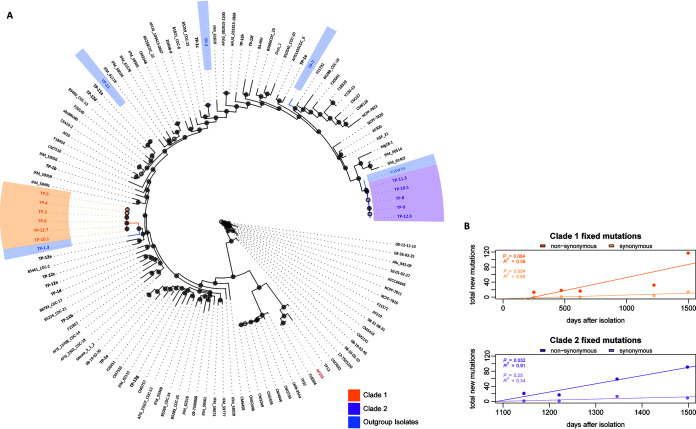
Whole-genome single nucleotide polymorphism phylogeny of 121 Aspergillus fumigatus isolates confirms genetic diversity and reveals 2 persistent clades in the AF100 series. (A) Illumina whole-genome sequencing and phylogenomic analyses generated a well-supported topology using 121 A. fumigatus isolates (29 from AF100). AF100 isolates are designated “TP-X.Y,” where X indicates the sample number and Y identifies different isolates from the same sample (where applicable). Clade 1 isolates are highlighted in orange, and clade 2 isolates are in purple. Isolates from outside these clades used for outgroup analysis are highlighted in blue, the AF293 reference is highlighted in red, and all AF100 isolates are listed in bold font. Filled circles at bipartitions represent >90% bootstrap support and open circles represent <90%. The data support a history of multiple colonization events for the AF100 patient, including both transient and persistent isolates. (B) Mutation accumulation curves for fixed synonymous and nonsynonymous mutations in the clade 1 and clade 2 isolates. Only those mutations acquired and then found in all subsequent isolates were considered in each clade. Significance was assessed using linear regression at a *P* value of <0.05 in the R programing environment. Clade 2 showed a significant accumulation of nonsynonymous mutations relative to synonymous mutations, suggesting clade 2 is under positive selection.

Genomic DNA from 29 AF100 series isolates (6 from AF100-1, 1 each from AF100-2 through AF100-9, 2 from AF100-10, 3 from AF100-11, and 10 from AF100-12) was sequenced with Illumina-based technology. Genomes were sequenced to an average depth of 55× coverage (see Table S1 in the supplemental material). To better understand the genetic relationships between the AF100 series isolates and to generate a phylogeny, we used the 29 genomes from the AF100 series and a collection of A. fumigatus genomes from non-CF clinical and environmental isolates (*n* = 92), including 68 publicly available genomes and 53 newly sequenced genomes. Across the 121 strains, we identified 223,369 variants relative to the AF293 reference genome (77,644 excluding intergenic, synonymous, and intronic variants). Of the total variants, 85,304 represent single nucleotide polymorphisms (SNPs), which were used to construct a 121-strain A. fumigatus maximum likelihood phylogeny that yielded a well-supported topology with most branches possessing >90% bootstrap support (Fig. 2A). AF100 isolates occupy ∼15 distinct positions across the tree, indicating the AF100 series represents a genetically diverse population.

10.1128/mBio.02153-21.6 TABLE S1 Whole-genome sequencing information on isolates in this study. Download Table S1, DOCX file, 0.1 MB. Copyright © 2021 Ross et al. 2021 Ross et al. https://creativecommons.org/licenses/by/4.0/ This content is distributed under the terms of the Creative Commons Attribution 4.0 International license.

While many isolates from the AF100 series occupy their own positions in the tree and do not group into a phylogenetic clade, 11 of the isolates cluster into two distinct clades, indicating repeated isolation of similar genotypes from the AF100 patient over the 4.5-year time course (Fig. 2A). Another isolate, TP-1.3, falls near but outside the larger of these clades. We designated these CF patient-specific clade 1 and clade 2. Both clades consist of isolates from time points that span large periods of time, with 6 isolates in clade 1 ranging from AF100-3 to AF100-12 (∼3.5 years) and 5 isolates in clade 2 ranging from AF100-8 to AF100-12 (∼1.5 years) (Fig. 2A; see also Fig. S1). The close genetic relationship between the isolates within each clade, as well as the timescale between isolations, supports the possibility that each clade represents repeated isolation of a persistent genotype from the AF100 individual. Furthermore, both clades feature isolates from the AF100-10 and AF100-12 plated sputum samples, which suggests they were present at the same time in the AF100 individual (Fig. 1A and 2A).

10.1128/mBio.02153-21.1 FIG S1 Single-colony images and normoxia growth measures for the isolates from clades 1 and 2. Briefly, 1 × 10^3^ conidia from the 7 isolates in clade 1 (A) or the 5 isolates in clade 2 (B) were grown on AMM plates for 72 h at 37°C under normoxia. (C) Colony diameter measurements were taken at 72 h, and significance was assessed via 1-way ANOVA with a Dunnett multiple-comparisons posttest, comparing to either TP-1.3 or TP-8 for clade 1 and 2, respectively. Download FIG S1, TIF file, 2.3 MB. Copyright © 2021 Ross et al. 2021 Ross et al. https://creativecommons.org/licenses/by/4.0/ This content is distributed under the terms of the Creative Commons Attribution 4.0 International license.

Although AF100 isolates that fell outside clades 1 and 2 were generally dispersed across the phylogeny, a single isolate from the AF100-1 sputum sample was most closely related to isolates of clade 1 (Fig. 2A). Isolation of TP-1.3 predated the first clade 1 isolate by ∼1 year (Fig. 1A), and considering the comparatively few variants that distinguish these two isolates (∼1,757 mutations) (Table S1), TP-1.3 may represent an ancestral isolate to clade 1. Although no clear ancestor was identified for clade 2 within the AF100 series, the environmental isolate IF1SW-F4 isolated from the International Space Station (37) surprisingly differs by only 643 total mutations (Fig. 2A; Table S1). These results indicate that the AF100 series represents a strikingly diverse population of A. fumigatus isolates, featuring at least 2 distinct genotypes that are either persistent or recurrent colonizers.

To further investigate the likelihood of these clades representing persistent genotypes compared to recurrent colonizers, we performed mutation accumulation analysis using the variant data from the different isolates of each clade. The analysis compared the number of fixed nonsynonymous variants in each clade over time to the number of fixed synonymous variants in each clade over time. A significant accumulation of nonsynonymous variants supports a given clade’s genotype as potentially being under positive selection, in this case, potentially facilitating persistence in the AF100 patient (38). Our analysis revealed a significant accumulation of nonsynonymous mutations in clade 2 but not in clade 1 (Fig. 2B). We consequently focused on clade 2 isolates to interrogate CF-relevant phenotypic characteristics of this A. fumigatus lineage.

### Clade 2 conidia have unique phenotypic profiles in response to CF-relevant stresses.

**FIG 3:**
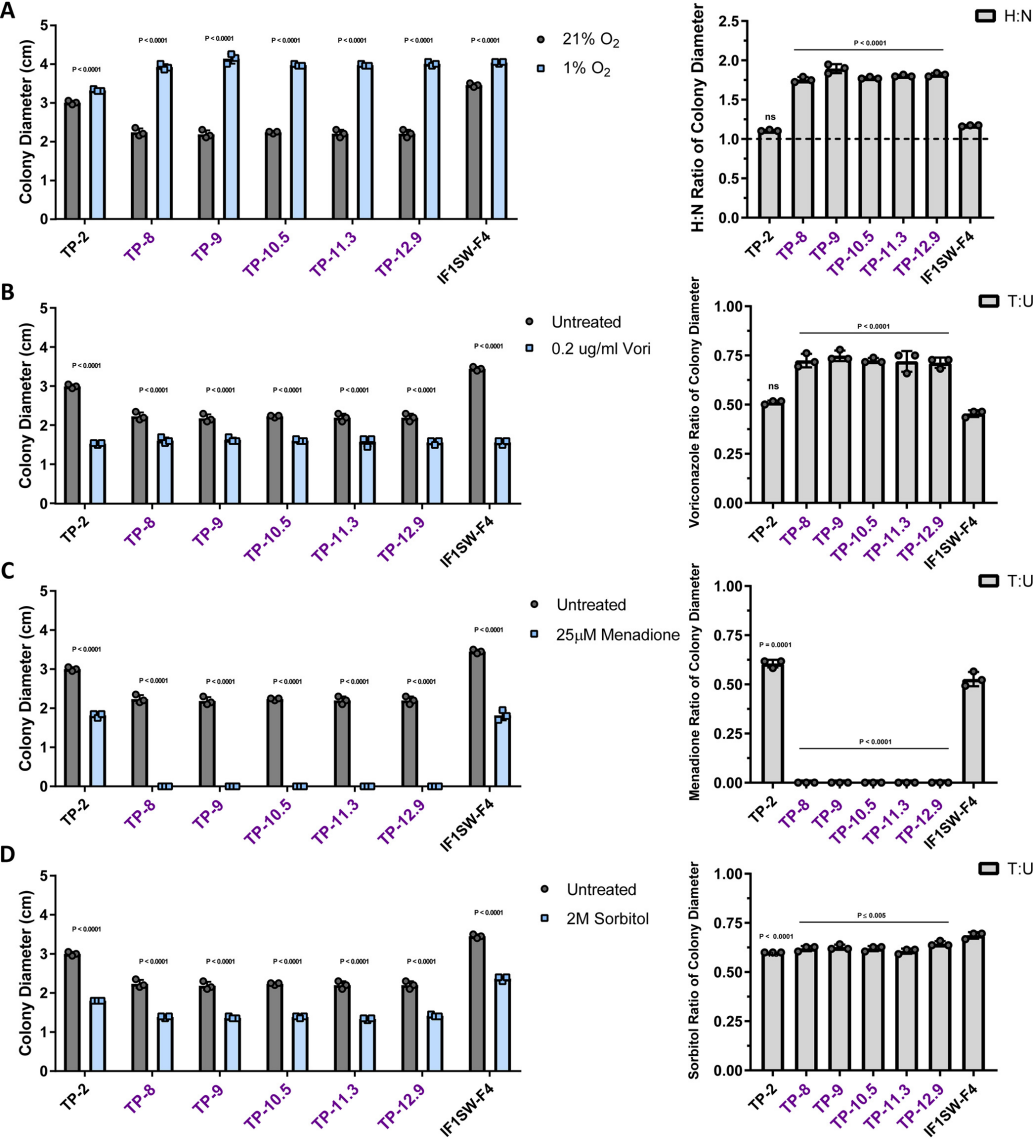
Clade 2 isolate conidia show altered CF-relevant stress phenotypes. Colony diameter was calculated by taking the average of perpendicular colony measurements, and hypoxia/normoxia or treated/untreated ratios (H:N and T:U, respectively) were calculated using these measurements. Each was calculated after conidial inoculation and 72 h of growth on AMM under hypoxic (1% O_2_) (A) sub-MIC voriconazole (0.2 μg/ml) (B) menadione-induced (25 μM) oxidative (C), and sorbitol-induced (2 M) osmotic (D) stress conditions, normalized to untreated growth. For colony diameter measurements, individual symbols represent the average colony diameter from 3 technical replicates. For H:N and T:U ratios, symbols represent the indicated ratio calculated per replicate, as assessed according to the described methods. Error bars represent standard deviations; for colony diameter, significance was assessed via 2-way ANOVA using the Šidák multiple-comparisons posttest to compare treated versus untreated for each isolate, and for H:N/T:U ratios, significance was assessed via 1-way ANOVA using a Dunnett multiple-comparisons posttest to compare all isolates to the IF1SW-F4 outgroup (using Prism 7; significance cutoff, *P* < 0.05). These findings indicate clade 2 isolates have altered CF-relevant stress resistance profiles relative to those of outgroups.

Under representative CF lung microenvironment conditions, clade 2 isolates had unique and adapted phenotypes compared to those of the control strains TP-2 (nonclade strain) and IF1SW-F4 (sequenced environmental strain). A striking phenotype for clade 2 isolates was reduced growth under normoxic conditions (21% O_2_, 5% CO_2_, 74% N_2_) compared to that under hypoxic conditions (1% O_2_, 5% CO_2_, 94% N_2_). Some clade 2 colonies grew nearly double in size under hypoxic conditions, resulting in hypoxia/normoxia growth ratios (H:N) ranging from ∼1.75 to 1.89 (Fig. 3A). Clade 2 isolates had significantly decreased growth under normoxic conditions compared to that under hypoxic conditions, as evidenced by these elevated H:N ratios compared to the those of the outgroup IF1SW-F4 (∼1.17) and TP-2 (∼1.10). When tested for growth in the presence of the triazole antifungal voriconazole, clade 2 isolates were significantly more resistant than outgroups, as evidenced by substantial colony growth on solid AMM containing 0.2 μg/ml voriconazole (Fig. 3B). Clade 2 voriconazole treated/untreated (T:U) ratio ranged from ∼0.71 to ∼0.75, significantly lower than those for TP-2 (0.51) and IF1SW-F4 (0.45) (Fig. 3B). All isolates had voriconazole MICs lower than the clinical resistance threshold as measured by CLSI broth microdilution (<1 μg/ml) (see Fig. S2) but were capable of growth on solid medium containing sub-MIC levels of drug. The finding that clade 2 conidia grow well at sub-MIC levels of voriconazole is particularly striking given that medical records indicate the AF100 patient has not received azole therapy since first being treated at DHMC (1996, ∼24 years).

10.1128/mBio.02153-21.2 FIG S2 MICs of voriconazole for a select group of isolates from this study. Standard microbroth dilution using voriconazole diluted in liquid AMM medium in 96-well plates was used, along with 1 × 10^5^ conidia per well from the indicated isolates. Plates were incubated for 24 h, and MIC was determined as the concentration of the first well with no visible fungal growth. Two biological replicates are shown. Download FIG S2, TIF file, 0.7 MB. Copyright © 2021 Ross et al. 2021 Ross et al. https://creativecommons.org/licenses/by/4.0/ This content is distributed under the terms of the Creative Commons Attribution 4.0 International license.

Conidia from clade 2 isolates were significantly more susceptible to the superoxide-generating compound menadione than TP-2 (T:U, 0.6) and IF1SW-F4 (T:U, 0.52) (Fig. 3C). Strikingly, clade 2 isolates were not able to germinate at the menadione concentration tested, a phenotype not shared with any other AF100 isolate (Fig. 3C). Conidia from clade 2 isolates were slightly yet significantly less resistant to sorbitol-mediated osmotic stress, with the clade 2 T:U ranging from 0.6 to 0.64 across the clade compared to 0.6 for TP-2 and 0.69 for IF1SW-F4 (Fig. 3D). Together, these results suggest that conidia from clade 2 isolates have unique phenotypes for CF-relevant stresses relative to those of reference strains, which suggests that clade 2 isolates may be uniquely adapted to the complex oxidative and osmotic environment found in CF airways (8).

### Comparative genomic analyses reveal mutations in stress response pathways that are unique to clade 2.

To identify alleles associated with the CF stress phenotypes observed in clade 2 isolates, we first utilized comparative genomic analyses. Compared to the AF293 reference genome, we identified 23,218 variants present in clade 2 isolates. Variant comparisons relative to the outgroup isolates (TP-2, TP-7, TP-11.1, and IF1SW-F4) reduced this set to 643 variants in clade 2. Because this method allows for possible miscalls and single deletions in various isolates, we further restricted the variant set to include only those appearing in all clade 2 isolates. This final set of 88 variants comprised 38 confident, conserved single-base deletions and 2 spanning deletions (see Data Set S1). To prioritize variants of interest that may be associated with specific clade 2 phenotypes, we further filtered the variants and identified 38 that were exclusive to clade 2 and found in no other isolates across the 121-isolate phylogeny. These clade 2 exclusive variants included 4 single nucleotide variant (SNV) deletions and 6 indels (see Data Set S2). Clade 2-exclusive variants were found in 37 unique genes, and one gene, Afu5g06915, had 2 variants. Interestingly, there were no overlapping variants exclusive to both clade 1 and clade 2 (found in both clades, but in no other isolates across the phylogeny).

10.1128/mBio.02153-21.8 DATA SET S1 Text file with list of variants found in all clade 2 isolates. Download Data Set S1, TXT file, 0.1 MB. Copyright © 2021 Ross et al. 2021 Ross et al. https://creativecommons.org/licenses/by/4.0/ This content is distributed under the terms of the Creative Commons Attribution 4.0 International license.

10.1128/mBio.02153-21.9 DATA SET S2 Text file of clade 2 exclusive variants. Download Data Set S2, TXT file, 0.1 MB. Copyright © 2021 Ross et al. 2021 Ross et al. https://creativecommons.org/licenses/by/4.0/ This content is distributed under the terms of the Creative Commons Attribution 4.0 International license.

Gene ontology (GO) term enrichment via the FungiDB database revealed that of the 37 genes with unique mutations in clade 2, six are annotated with roles in stress sensing and response: Afu1g07160 (*ubp3*), Afu1g15950 (*pbs2/B*), Afu2g10620, Afu3g05900 (*ste7*), Afu3g08010 (*sltA/ace1*), and Afu3g11940 (39). Specifically, Pbs2 and Ste7 are well-studied MAPK kinases (MAPKKs) involved in extracellular stress responses. Pbs2 has roles in response to hypoxic, oxidative, and osmotic stresses as part of the well-studied HOG MAPK pathway in fungi (40–42); Ste7 is the MAPKK of the mating/invasive growth (MpkB) pathway and is responsive to nutrient conditions and the presence of other fungi (43). Ubp3 and AceI/SltA have been demonstrated to interact with Ste7 (in other fungal species) and downstream MpkB pathway members, respectively; AceI/SltA is involved in salt stress and is responsive to voriconazole (44–47). Afu3g10080 and Afu3g11970 (*pacC*) are also mutated uniquely in clade 2 and have annotated roles in response to intracellular stress; the latter gene is a known virulence determinant and internal pH stress-response factor and works concomitantly with AceI/SltA (48). These 8 genes and their mutations of interest are summarized in Table 1. Given the oxidative and osmotic stress phenotypes observed in clade 2 isolates and the association of the HOG MAPK pathway with these stress responses, the cytosine-to-thymine point mutation at position 590 in the coding sequence of *pbs2* (*pbs2^C2^*) was investigated further.

**TABLE 1:** Genes and mutations of interest from clade 2 isolates

Gene ID^*a*^	Product description (FungiDB)	Type of mutation	Nucleotide change
Afu1g07160 (*ubp3*)	Ortholog(s) has ubiquitin-specific protease activity, role in protein deubiquitination and cytosol, nucleus localization	Splice donor variant	522 + 2T→G
Afu1g15950 (*pbs2*)	Putative mitogen-activated protein kinase (MAPKK)	Missense variant	590C→T
Afu2g10620	Ortholog(s) has protein serine/threonine kinase activity, protein serine/threonine kinase inhibitor activity	Missense variant	199G→A
Afu3g05900 (*ste7*)	MAPK kinase (MAPKK)	Start site lost	2T→C
Afu3g08010 (*ace1*)	C2H2 transcription factor	Missense variant	2175G→C
Afu3g10080	Ortholog(s) has cytosol localization	Missense variant	531C→G
Afu3g11940	Has domain(s) with predicted zinc ion binding activity	Stop codon gained	1711C→T
Afu3g11970 (*pacC*)	C2H2 finger domain transcription factor	Frameshift variant	1628dupC

a ID, identifier.

### The *pbs2^C2^* allele is necessary for CF-relevant stress phenotypes in TP-9 conidia.

**FIG 4:**
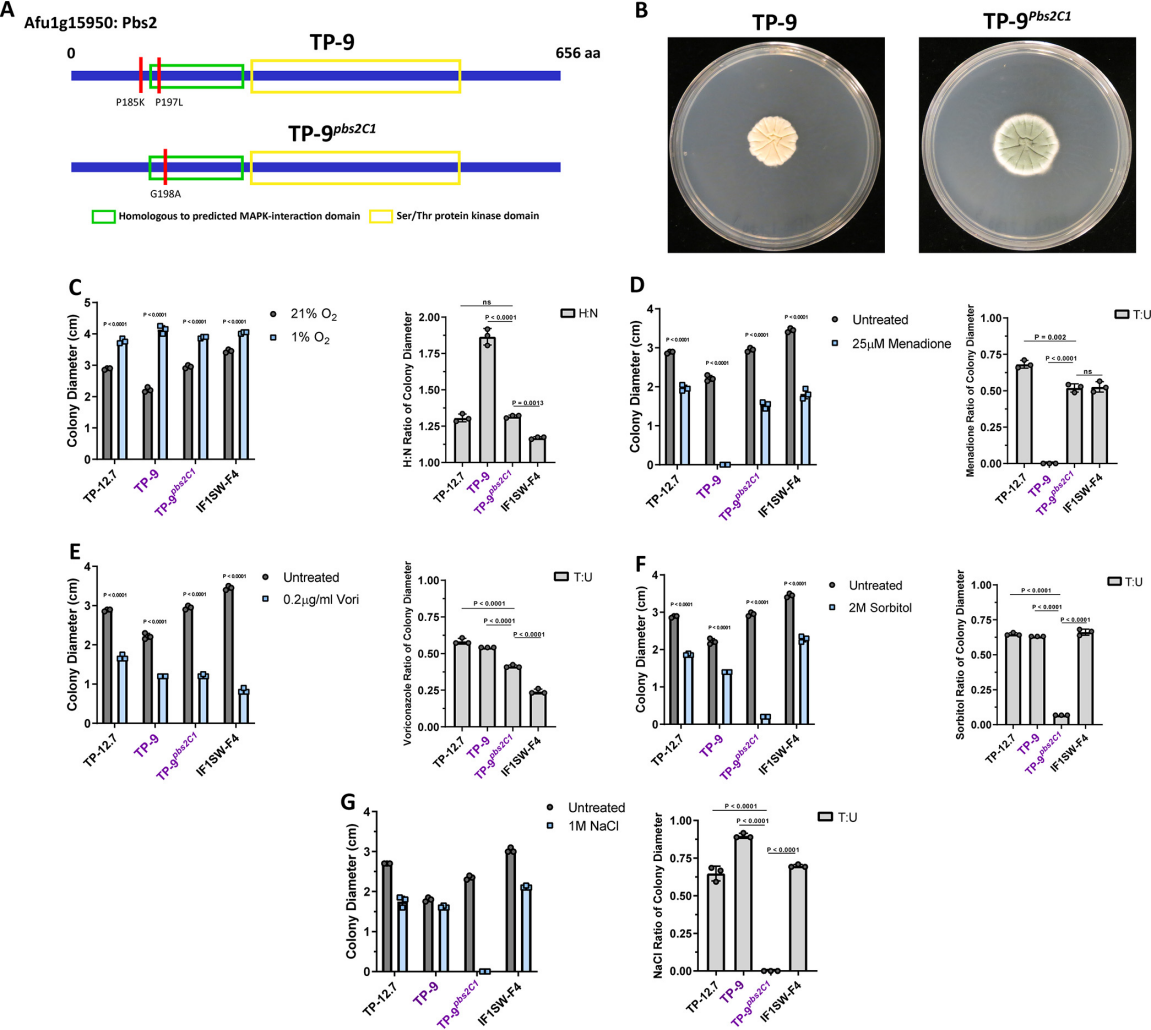
Allelic replacement of *pbs2^C2^* to generate TP-9*^pbs2C1^* results in morphological changes and significantly altered conidial stress responses. (A) Schematic representation of the Pbs2 protein domains in A. fumigatus, with variants in each of the indicated isolates marked in red (4 total nucleotide changes). (B) TP-9*^pbs2C1^* displays morphological and growth changes under normal conditions (72 h at 37°C under normoxia). For conidial phenotyping, symbols represent the average H:N and T:U ratios, as described previously. For colony diameter, significance was assessed via 2-way ANOVA using a Šidák multiple-comparisons posttest to compare treated versus untreated for each isolate, and for H:N and T:U ratios, significance was assessed via 1-way ANOVA using a Dunnett multiple-comparisons posttest to compare all isolates to the TP-9*^pbs2C1^* mutant (Prism 7; significance cutoff, *P* < 0.05). TP-9*^pbs2C1^* displayed significantly altered profiles for the clade 2 oxygen phenotypes, including partially rescued oxygen sensitivity under both hypoxia (C) and oxidative stress (D) conditions. (E) Interestingly, TP-9*^pbs2C1^* showed a slight decrease in voriconazole resistance. (F) Most notably, the TP-9*^pbs2C1^* mutant showed a significant growth defect in the presence of osmotic stress, suggesting the *pbs2^C2^* allele may be important for persistence of clade 2.

The 590 point mutation is one of 2 nonsynonymous SNPs in Pbs2^C2^ compared to the AF293 reference genome, and the allele results in a unique P197L amino acid substitution in a region homologous to a domain necessary for MAPK interaction in Saccharomyces cerevisiae (Fig. 4A) (49). To test the hypothesis that *pbs2^C2^* is necessary for the observed clade 2 phenotypes, we conducted an allelic exchange experiment using the clade 2 isolate TP-9 and the *pbs2* allele from clade 1 isolate TP-12.7 to generate strain TP-9*^pbs2C1^* (*pbs2^C1^*) (Fig. 4A). The TP-9*^pbs2C1^* strain was confirmed via Sanger sequencing and Southern blot analysis (Fig. S5). As predicted, the TP-9*^pbs2C1^* mutant conidia had a significantly altered stress resistance profile relative to that of the TP-9 background (Fig. 4B to E). TP-9*^pbs2C1^* grew significantly better than TP-9 under normoxic conditions, as reflected by the smaller H:N ratio (∼1.31 versus ∼1.86, respectively), and also phenocopied TP-12.7, the source of the *pbs2^C1^* allele (Fig. 4C). Significant conidiation was also restored in TP-9*^pbs2C1^* (Fig. 4B). Additionally, TP-9*^pbs2C1^* conidia were capable of growth on menadione in contrast to TP-9, with an overall higher T:U ratio (Fig. 4D); this growth in the presence of menadione is even more striking considering TP-9 conidia do not germinate in the presence of menadione (Fig. 3C). Together with the hypoxia/normoxia colony growth, these data indicate that the *pbs2^C2^* allele is necessary for the observed oxygen sensitivity of clade 2 conidia. Considering the above-described change in oxygen sensitivity and the importance of oxygen in ergosterol biosynthesis, we also examined the voriconazole resistance of the TP-9*^pbs2C1^* mutant. As expected, TP-9*^pbs2C1^* showed a decrease in voriconazole resistance relative to that of the no-drug control and TP-9 (Fig. 4E).

10.1128/mBio.02153-21.4 FIG S4 Representative images (A) and raw fluorescence signal data (B) for quantitative Western blotting to detect the total and phospho-SakA-FLAG levels in the indicated strains. Using the LI-COR Odyssey CLx system, images were recorded using the manufacturer’s IRDye 680RD goat anti-mouse IgG and IRDye 800CW goat anti-rabbit IgG at 1:10,000 dilution to detect SakA-FLAG and P-SakA-FLAG, respectively. Antibody specificity was confirmed using the Δ*sakA* mutant and untransformed TP-9 isolates as controls. Download FIG S4, TIF file, 1.2 MB. Copyright © 2021 Ross et al. 2021 Ross et al. https://creativecommons.org/licenses/by/4.0/ This content is distributed under the terms of the Creative Commons Attribution 4.0 International license.

10.1128/mBio.02153-21.5 FIG S5 Southern blot using gDNA from TP-9, TP-9^*pbs2C1*^, and another TP-9^*pbs2C1*^ transformant from the same experiment. Primers RAC 4632 and RAC 4633 and the PCR DIG Probe Synthesis Kit (Roche Diagnostics, Mannheim, Germany) were used to generate the DIG-labeled DNA probe, and the PstI endonuclease was used for restriction digest of the gDNA prior to hybridization as previously described (81). The farthest lane (left to right) contains the DIG-labeled Molecular Weight Marker VII (Roche) for estimation of fragment sizes. Based on the insertion of the *ptrA* pyrithiamine resistance sequence as part of the *pbs2* allelic exchange, the resulting hybridized fragment in the TP-9^*pbs2C1*^ transformants is larger than TP-9 by ∼2 kb. All strains share a band at ∼2000 bp based on their shared genetic background. FIG S5, TIF file, 1.6 MB. Copyright © 2021 Ross et al. 2021 Ross et al. https://creativecommons.org/licenses/by/4.0/ This content is distributed under the terms of the Creative Commons Attribution 4.0 International license.

We lastly examined the osmotic stress resistance of the TP-9*^pbs2C1^* strain conidia compared to that of TP-9. We expected to observe an increase in osmotic stress resistance in TP-9*^pbs2C1^* strain conidia based on the oxidative stress phenotype and known contributions of the HOG pathway to both osmotic and oxidative stress responses. However, TP-9*^pbs2C1^* conidia showed a severe growth defect in the presence of 2 M sorbitol, with slower growth in the treated condition; the T:U ratio for TP-9*^pbs2C1^* (∼0.07) was significantly lower than those of TP-9, TP-12.7, and IF1SW-F4, the non-CF isolate closely related to clade 2 (Fig. 4F). To confirm this phenotype was not sorbitol specific, we also used AMM with or without 1 M NaCl and observed the same effect (Fig. 4G); in fact, NaCl showed an even stronger effect and prevented TP-9*^pbs2C1^* conidial germination. The conidia osmotic stress susceptibility in the absence of *pbs2^C2^* suggests that the *pbs2^C2^* allele confers increased conidial osmotic stress resistance in the CF lung environment at the cost of reduced oxidative stress resistance. Taken together, the phenotypic profiling data indicate the *pbs2^C2^* allele is necessary for the unique CF-relevant conidial phenotypes found in clade 2.

### Pbs2^C2^ is not associated with menadione susceptibility in hyphae but is necessary for osmotic resistance independent of growth stage.

**FIG 5:**
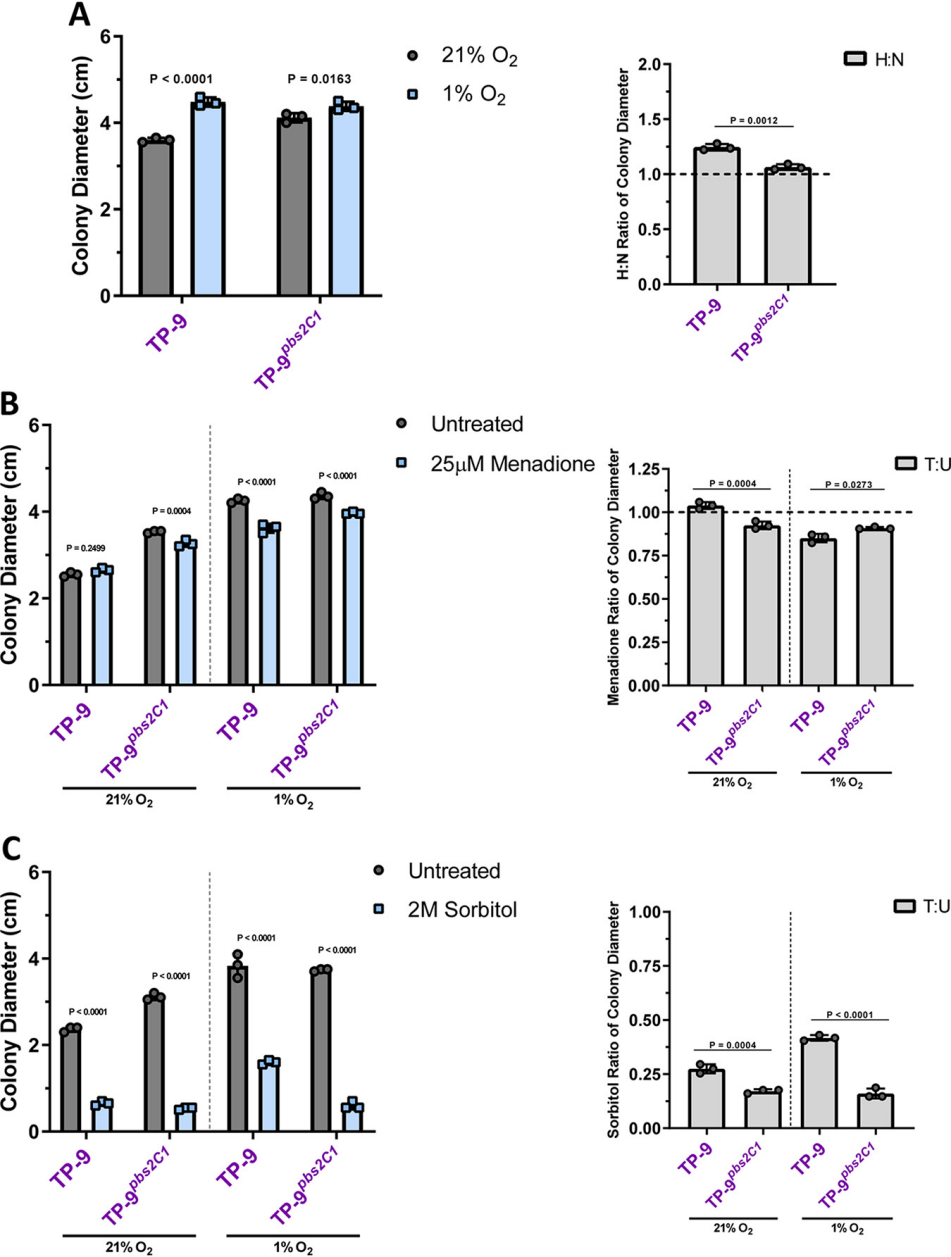
*pbs2^C2^* is not associated with severe oxidative stress susceptibility in hyphae but is necessary for osmotic stress resistance. Hyphal phenotypic stress assays used 0.5-cm^2^ hyphal plugs cut from 48-h cultures and grown for an additional 72 h at 37°C. Individual symbols represent the average colony diameter and the average H:N/T:U ratios, as described previously. For colony diameter, significance was assessed via 2-way ANOVA using a Šidák multiple-comparisons posttest to compare treated versus untreated for each isolate, and for H:N and T:U ratios, significance was assessed via 1-way ANOVA using a Tukey multiple-comparisons posttest to compare all isolates based on oxygen conditions (using Prism 7, significance cutoff *P* < 0.05). TP-9 hyphae had a lower H:N ratio (A) and a lower T:U ratio (B) than conidia under oxidative stress conditions, but similar trends held up compared to TP-9*^pbs2C1^* hyphae. (C) Osmotic stress conditions resulted in poor growth for TP-9*^pbs2C1^* compared to that of TP-9, suggesting *pbs2^C2^* is necessary for resistance.

The severe clade 2 isolate oxygen sensitivity presented a conundrum if these were isolates persisting in the CF lung. How could these isolates evade the robust oxidative burst utilized by leukocytes to prevent A. fumigatus infection and progress disease? We thus considered that hyphal stress responses may reveal more about the importance of the *pbs2^C2^* allele in A. fumigatus persistent CF infections. We modified the conidial stress assay to use 0.5-cm hyphal plugs from established colonies as the starting inoculum. Similar to the observed normoxia growth phenotype with conidia, TP-9 hyphae had a significantly higher H:N ratio than TP-9*^pbs2C1^* (Fig. 5A). This result indicates that, as with conidia, the TP-9 hyphae have reduced growth under normoxia; however, the magnitude of this phenotype was not as large as that observed with conidia (Fig. 4A and 5A). Surprisingly, however, TP-9 hyphae were unaffected by menadione under normoxia and, under hypoxia, only showed a minor reduction in growth (Fig. 5B). This result with TP-9 hyphae is in striking contrast to TP-9 conidia that did not germinate at the same menadione concentration. Despite the comparable resistance of the TP-9*^pbs2C1^* conidia to menadione, the TP-9*^pbs2C1^* hyphae were slightly more susceptible than TP-9 hyphae under normoxia and slightly more resistant under hypoxia (Fig. 5B). Thus, while the *pbs2^C2^* allele alters the response to menadione in hyphae, the effect size between TP-9 and TP-9*^pbs2C1^* suggests that the *pbs2^C2^* allele is not directly necessary for superoxide-mediated stress resistance in TP-9 hyphae (Fig. 5B). These data support the conclusion that the *pbs2^C2^* allele is more detrimental to conidial responses to oxygen stress than to responses in mature hyphae.

We next examined the osmotic stress sensitivity of TP-9 hyphae. Regardless of oxygen conditions, TP-9 hyphae were significantly more resistant to osmotic stress via sorbitol than TP-9*^pbs2C1^* hyphae (Fig. 5C). These data suggest *pbs2^C2^* is necessary for osmotic stress resistance in TP-9 regardless of fungal growth stage. The magnitude of the osmotic stress sensitivity in TP-9*^pbs2C1^* hyphae was larger under hypoxia than under normoxia, further suggesting that the *pbs2^C2^* allele is important for clade 2 stress responses under CF-like conditions. Overall, these results strongly suggest that the *pbs2^C2^* allele results in increased hyphal stress resistance at the cost of conidial oxygen sensitivity in clade 2 and that osmotic stress and low-oxygen conditions are important factors in the CF lung environment that impact A. fumigatus persistence.

### The *pbs2^C2^* allele is not sufficient to replicate clade 2 stress phenotypes in other isolates.

We next asked if the *pbs2^C2^* allele is sufficient to induce the unique clade 2 oxidative and osmotic stress phenotypes in another genetic background. We chose TP-12.7, as it is the most recent isolate from clade 1 and is the source of the *pbs2^C1^* allele. We performed this allelic exchange according to the same construct design using *pbs2^C2^* to generate the TP-12.7*^pbs2C2^* strain. We used conidia from TP-12.7 and the TP-12.7*^pbs2C2^* strain to examine growth under hypoxic, oxidative, and osmotic stress conditions. There was no significant difference between TP-12.7 and the TP-12.7*^pbs2C2^* strain for hypoxic growth with conidia or hyphae (see Fig. S3A). Interestingly, the extreme clade 2 conidial menadione sensitivity phenotype was also not observed in TP-12.7*^pbs2C2^*, indicating that the *pbs2^C2^* allele is not sufficient to induce conidial menadione susceptibility in the clade 1 genetic background (Fig. S3B). Furthermore, there was no significant difference between the strains for osmotic stress, indicating this allele is not sufficient for the clade 2 growth phenotypes in at least the TP-12.7 genetic background (Fig. S3C).

10.1128/mBio.02153-21.3 FIG S3 Conidial phenotyping of the TP-12.7*^pbs2C2^* (A to C) and the IF1SW-F4*^pbs2C2^* (D to G) mutants reveals *pbs2^C2^* is not sufficient to reproduce the clade 2 phenotypes. TP-12.7*^pbs2C2^* showed no significant difference in H:N ratio (A) or T:U ratio (C) under osmotic stress conditions and was actually more resistant to menadione (B) than TP-12.7. Using the more closely related IF1SW-F4 background, we also saw no significant difference in H:N ratios between IF1SW-F4 and IF1SW-F4*^pbs2C2^* (D) and no difference for T:U ratios for menadione under hypoxic conditions (E), sorbitol under normoxic conditions (G), or voriconazole under either oxygen condition (F). Although IF1SW-F4*^pbs2C2^* had a significantly larger T:U ratio for menadione under normoxia (E) and sorbitol under normoxia, neither phenotype was found in slade 2, indicating *pbs2^C2^* is not sufficient to replicate clade 2 stress phenotypes. Assays and statistical analyses were performed as described for Fig. 5. Download FIG S3, TIF file, 1.7 MB. Copyright © 2021 Ross et al. 2021 Ross et al. https://creativecommons.org/licenses/by/4.0/ This content is distributed under the terms of the Creative Commons Attribution 4.0 International license.

To understand the sufficiency of the *pbs2^C2^* allele in an additional genetic background, we also performed the allelic exchange experiment in the closely related IF1SW-F4 strain. As the clade 2 outgroup, this isolate is more closely related to clade 2 but does not share its unique phenotypic characteristics. We used the same construct design as detailed previously, using the *pbs2^C2^* sequence to generate the IF1SW-F4*^pbs2C2^* strain. Based on the overall larger magnitude of the conidial phenotypes associated with *pbs2^C2^* in clade 2, we used conidia from the IF1SW-F4 and IF1SW-F4*^pbs2C2^* strains to examine growth under hypoxic, oxidative, osmotic, and voriconazole stress conditions.

No significant difference between IF1SW-F4 and the IF1SW-F4*^pbs2C2^* strains was observed under low-oxygen growth conditions (Fig. S3D). Furthermore, there was no significant difference between these isolates with voriconazole or osmotic stress under normoxia (Fig. S3F and G). This was true for voriconazole under hypoxia as well, although the IF1SW-F4*^pbs2C2^* mutant was slightly more resistant to osmotic stress under hypoxia (Fig. S3F and G). We also found IF1SW-F4*^pbs2C2^* to be slightly more resistant under menadione stress under normoxia (Fig. S3E). Despite this, neither of these phenotypes match those of clade 2, indicating the *pbs2^C2^* allele is also not sufficient to induce clade 2 phenotypes in the IF1SW-F4 background. These data suggest genetic interactions with other allelic variants in the clade 2 background are necessary to fully express the unique clade 2 Pbs2-mediated stress responses.

### Pbs2^C2^ is necessary for full HOG pathway activity in response to osmotic stress in clade 2.

**FIG 6:**
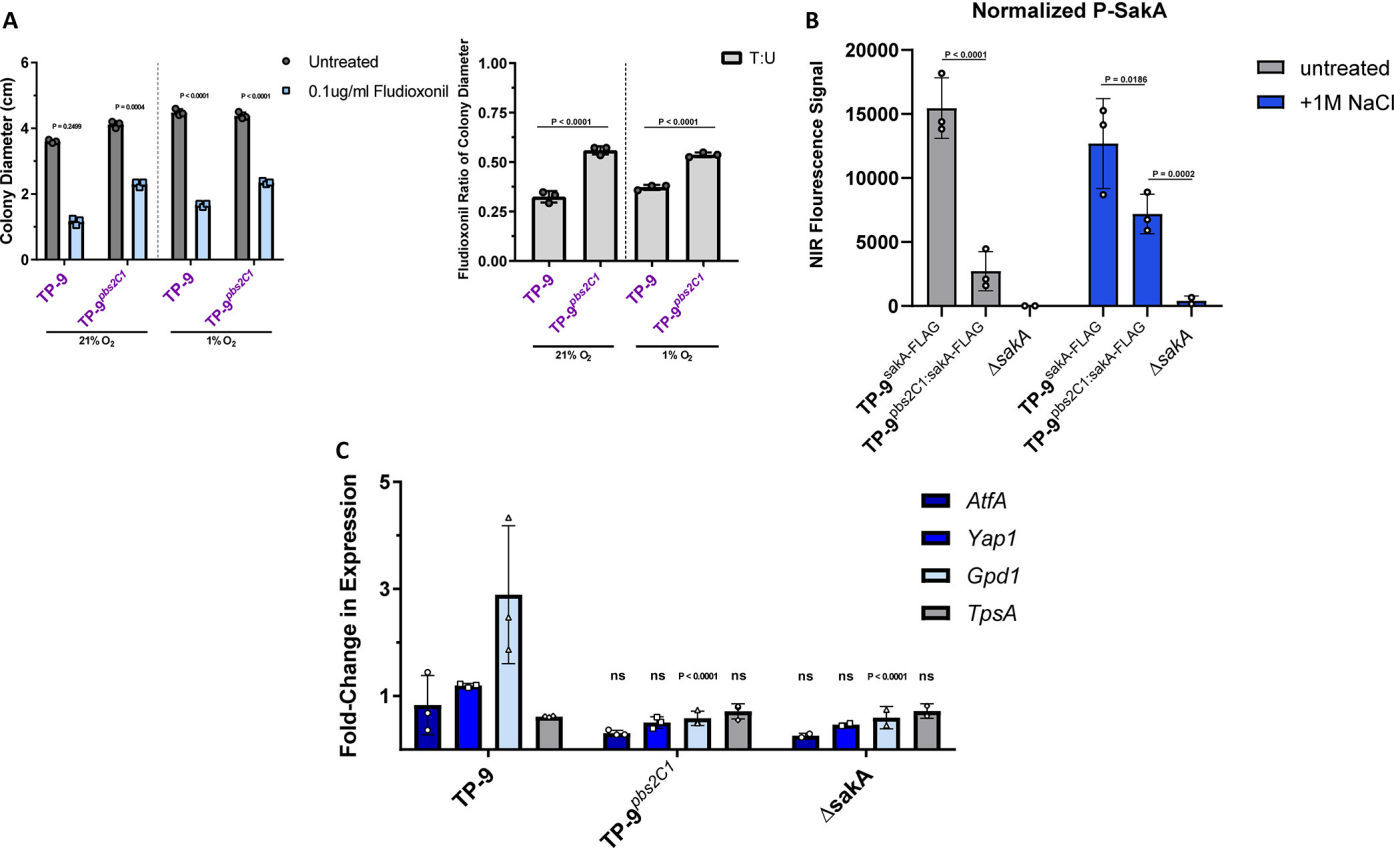
Loss of *pbs2^C2^* results in reduced HOG pathway activity in response to osmotic stress. (A) Using the hyphal stress assay as previously described, TP-9*^pbs2C1^* was significantly more resistant to growth on AMM plus 0.1 μg/ml fludioxonil, which suggests reduced HOG pathway activity. For colony diameter, significance was assessed via 2-way ANOVA using a Šidák multiple-comparisons posttest to compare treated versus untreated for each isolate, and the T:U ratio was assessed via 1-way ANOVA using a Tukey multiple-comparisons posttest to compare isolates based on oxygen conditions (using Prism 7; significance cutoff, *P* < 0.05). (B) Quantification from LI-COR fluorescent Western blotting for P-SakA (the active form of the Pbs2 target). Bars represent the averages from 3 biological replicates (symbols), normalized to the REVERT total protein stain protocol (LI-COR Biosciences). Statistical analysis was performed using 2-way ANOVA with a Dunnett multiple-comparisons posttest to compare all isolates to TP-9 under the same conditions, and error bars represent standard deviations. TP-9*^pbs2C1^* has lower P-SakA levels under both untreated and 1 M NaCl conditions, which suggests that the *pbs2^C2^* allele is involved in altering HOG pathway activity. (C) mRNA levels of HOG pathway-associated target genes. *pbs2^C2^* qRT-PCR was performed using primers for the actin (*actA*) and β-tubulin (*tubA*) housekeeping genes as well as a selection of downstream SakA (HOG) targets (*atfA*, *yap1*, *gpd1*, and *tpsA*). RNA was extracted from mycelia in mid-log phase transferred to fresh AMM or AMM plus 1 M NaCl for 15 min. Bars represent the averages from 3 biological replicates (symbols) normalized to the fresh AMM group. Statistical analysis was performed using 2-way ANOVA with a Dunnett multiple-comparisons posttest to compare all isolates to TP-9 on a transcript-by-transcript basis; significance cutoff, *P* < 0.05. ns, *P* > 0.13. The lower response by TP-9*^pbs2C1^* suggests it has reduced SakA signaling in response to osmotic stress.

In order to understand the effect of the clade 2 *pbs2* allele on HOG pathway function, we first tested our isogenic set of strains for sensitivity to the drug fludioxonil. In fungi, resistance to fludioxonil requires loss of HOG pathway activity (50, 51). We observed that TP-9 was more susceptible to fludioxonil than TP-9*^pbs2C1^* under both normoxic and hypoxic conditions, which suggests that TP-9 has increased HOG pathway activity relative to that of TP-9*^pbs2C1^* (Fig. 6A). To further test if *pbs2^C2^* is required for HOG pathway activity in response to stress, we examined the phosphorylation of SakA, the A. fumigatus HOG MAPK homolog, via quantitative fluorescence Western blotting using the LI-COR Odyssey imaging platform. To perform 2-color pan/phospho-SakA detection, we first generated TP-9 and TP-9*^pbs2C1^* strains containing a 3×FLAG-tagged *sakA* allele (Fig. S4A). In these strains, the *sakA* locus was replaced with a *sakA*:*3×FLAG* allele. These strains phenocopied TP-9 and the TP-9*^pbs2C1^*, indicating the presence of functional SakA with the FLAG epitope tag.

Using stationary liquid cultures that were shifted to either fresh AMM or AMM plus 1 M NaCl, we observed that, relative to that in the TP-9*^pbs2C1^*^:sakA-FLAG^ strain, TP-9*^sakA^*^-FLAG^ had significantly more phospho (P)-SakA signal upon NaCl induced osmotic stress (Fig. 6B; Fig. S4B). Furthermore, TP-9*^sakA^*^-FLAG^ also showed high P-SakA signal in the absence of stimulus. These data support the hypothesis that *pbs2^C2^* results in increased HOG pathway activity in clade 2 isolates (Fig. 6B; Fig. S4B). These data suggest a molecular basis for the oxidative resistance phenotypes in TP-9 relative to the phenotypes in the related environmental isolate IF1SW-F4.

To further test our hypothesis that *pbs2^C2^* is necessary for increased HOG pathway activity in clade 2, we examined expression of 4 putative SakA (HOG) target genes in response to NaCl stress (52). The HOG target genes were chosen based on their roles in response to different stresses: Afu3g11330 (*atfA*), a bZIP transcription factor required for oxidative and heat stress resistance, Afu6g09930 (*yap1*), a key regulator of the oxidative stress response, Afu2g08250, a homolog of *gpd1* necessary for glycerol biosynthesis and osmotic stress resistance in S. cerevisiae, and Afu6g12950 (*tpsA*), which is required for trehalose biosynthesis in response to temperature stress (53–56).

TP-9 hyphae were able to mount a strong transcriptional response to osmotic shock, as indicated by increased mRNA levels of *gpd1* (Fig. 6C). The specificity and necessity of this response for osmotic stress resistance is shared across a number of fungal species (57). However, loss of the *pbs2^C2^* allele resulted in significantly reduced *gpd1* induction and no change in other examined HOG pathway target gene mRNA levels. This low *gpd1* mRNA level was similar to levels observed in the Δ*sakA* control strain, further suggesting TP-9*^pbs2C1^* has reduced HOG pathway activity compared to that of the CF isolate TP-9 in response to NaCl (Fig. 6C). Taken together, the unique *pbs2^C2^* allele is necessary for clade 2 conidial oxidative stress susceptibility and growth stage-independent osmotic stress resistance via increased HOG pathway activity.

## DISCUSSION

Aspergillus fumigatus colonization in CF is a growing concern, as recent studies and better diagnostic approaches indicate a greater degree of prevalence than previously appreciated (16, 58). An outstanding question is how A. fumigatus persistence in the CF lung contributes to disease progression. Previous studies of CF bacterial isolates identified substantial genetic diversity and specific mutations that drive pathogenesis in the CF airways (reviewed in references 24 and 59); these studies have greatly increased our knowledge of CF bacterial pathogenesis mechanisms and rationale for empirical antibiotic therapies. In contrast, far less is known about the genotypic and phenotypic diversity of CF A. fumigatus isolates. A greater understanding of A. fumigatus colonization, persistence, and pathogenesis mechanisms is expected to help clarify when and if to apply antifungal therapies in CF patients. Here, we observed both transitory and persistent A. fumigatus isolates from sputum samples of a CF patient collected longitudinally over 4.5 years. Phenotypic analyses of the persistent isolates revealed increased fitness in response to osmotic stress and decreased fitness in ambient oxygen concentrations compared to those in the closely related environmental isolate IF1SW-F4. Genotypic and genetic analyses discovered an important role for the HOG MAP kinase pathway via novel mutations in the MAPKK *pbs2*, which drove the emergence of these stress response phenotypes. These data support hypotheses that CF patients can be persistently infected with specific A. fumigatus genotypes and that the fungus acquires adaptive mutations to promote fitness in the dynamic CF lung environment (60, 61).

Our genomic analyses of the AF100 patient A. fumigatus isolates are consistent with previous studies of A. fumigatus intraspecies diversity. Specifically, CF patients may be colonized by more than one A. fumigatus genotype over time (62). Genomic analysis of the A. fumigatus isolates from the AF100 patient revealed extensive genetic diversity among the 29 isolates that occupy ∼15 distinct positions in the phylogeny of 121 isolates (Fig. 2). Genetic differences between individual isolates in our study ranged from a few hundred to tens of thousands of variants. These data lend support to the long-standing hypothesis that numerous A. fumigatus genotypes are capable of colonizing the CF lung (35, 62). However, an important consideration for our and previous studies is that we cannot rule out that genotypes which appeared one time in our longitudinal sampling are only transitory colonizers or environmental contaminants. These data emphasize the importance of repeated isolation of specific related genotypes in informing a diagnosis of a potential infection (and thus treatment) versus transitory colonization or environmental contamination.

In the present study, two genotypically distinct clades of persistent isolates were identified in the patient living with CF. Intriguingly, evidence for positive selection was only observed in the clade 2 isolates. We cannot rule out that clade 1 isolates previously experienced a positive selection event prior to our sampling or are newer to the CF patient lung environment. Further studies of clade 1 isolates are needed to better understand their genotypic and phenotypic profiles, but it is also possible that clade 1 and 2 isolates occupy distinct microenvironments in the CF lung. A significant accumulation of fixed nonsynonymous mutations in clade 2 over time suggests selective pressure from persistence in the CF lung environment (38). Moreover, compared to the closely related environmental isolate IF1SW-F4, clade 2 isolates had significantly increased fitness to CF-relevant osmotic stress and a preference for growth in a low-oxygen atmosphere in addition to increased antifungal drug resistance (8, 63). These phenotypes are consistent with a genotype that has been shaped in part by long-term persistence in the CF lung environment.

The MAPK pathway is known to mediate fungal cellular responses to hypoxic, oxidative, antifungal, and osmotic stresses, among others (64–67). Moreover, HOG pathway mutation in clinical fungal isolates was previously documented and associated with altered stress responses (41, 68). Consequently, the unique *pbs2^C2^* allele found only in clade 2 isolates was chosen to interrogate its role in the observed clade 2 isolate phenotypes in this study. Through allelic exchange experiments, we observed that *pbs2^C2^* is necessary for HOG pathway signaling in response to osmotic stress in both conidia and hyphae of clade 2 isolates. Given the relatively ubiquitous nature of osmotic stress in CF mucus plugs, it follows that osmotic stress acts as a strong selective pressure in CF A. fumigatus initial colonization and long-term persistence (69).

Additional evidence that the *pbs2^C2^* allele arose *in vivo* in response to the CF lung environment is suggested by the conidium- and hypha-specific phenotypes of clade 2 isolates. For example, clade 2 conidia were extremely susceptible to menadione-induced oxidative stress and this was dependent on the *pbs2^C2^* allele (Fig. 4). In contrast, the lack of severe oxidative stress sensitivity in clade 2 hyphae is likely more relevant than the conidial phenotype in the context of CF lung persistence (Fig. 5). A number of studies have reported visualization of hyphae and hyphal fragments in expectorated sputum from CF patients, which suggests certain isolates grow and develop in the CF lung environment (70, 71). Furthermore, conidial production is an energetically costly process and typically does not occur *in vivo* unless in a pulmonary cavity (72). Lastly, although aerosol transmission of A. fumigatus between CF patients has been reported, it is unknown whether this is predominantly aerosolized hyphal fragments or conidia (29). Taken together, these data suggest the enhanced osmotic stress resistance of clade 2 isolates comes at the expense of conidial fitness and thus likely arose *in vivo.*

Clade 2 isolates also displayed a significant growth reduction under ambient atmospheric oxygen conditions that was also *pbs2^C2^* dependent. This observation has potential clinical ramifications when it comes to culture of sputum or bronchial lavage samples from CF patients. Given the reduced growth rate of these CF lung-adapted isolates compared to that of the nonpersistent isolates and the clinical microbiology practice of growing fungal cultures under atmospheric oxygen conditions, we speculate that persistent A. fumigatus isolates could be missed in clinical samples. Moreover, it was clear from our sputum sampling that picking a single A. fumigatus colony from a sputum plate may not reflect the true genotypic and phenotypic potential of isolates in the airways of a given patient with CF. Further studies are needed to test this hypothesis which has significant ramifications for detecting A. fumigatus infections in CF patients and in other suspected manifestations of aspergillosis (73).

Molecular details on how the specific P197L mutation in Pbs2^C2^ promotes increased fitness in the CF lung environment remain to be determined. However, the P197L mutation occurs in a region homologous to a predicted MAPK interaction domain in S. cerevisiae (49). Although this region of Pbs2 has not been shown to be involved in upstream or downstream HOG MAPK signaling in A. fumigatus, our data with fludioxonil strongly suggests the former. Fludioxonil antifungal activity in A. fumigatus is dependent on the type-III hybrid histidine kinase (HHK) TcsC (51). One of a number of putative histidine kinases that can activate the HOG pathway in A. fumigatus, TcsC signals specifically through the Ypd1-Ssk2/22-Ssk1 arm of the HOG pathway (40). Because loss of *pbs2^C2^* results in fludioxonil resistance in clade 2 isolates, Pbs2^C2^ likely promotes HOG pathway activation through the TcsC-Ypd1-Ssk2/22/1 arm. However, we cannot rule out unique genetic interactions with other observed signaling mutations in the clade 2 strains. In support of this alternative hypothesis, the *pbs2^C2^* allele was not sufficient to promote increased osmotic stress resistance or conidial oxidative stress sensitivity in the clade 1 or closely related IF1SW-F4 genetic background (see Fig. S3 in the supplemental material). Thus, additional genetic interactions involving the *pbs2^C2^* allele in the clade 2 background await further investigations. For example, the presence of variants associated with *ste7* in clade 2 isolates is an intriguing observation for further study and likely evidence of cross talk between these 2 MAPK pathways.

An important and unanswered question raised by this single-patient longitudinal study is whether the unique mutation in Pbs2 or other HOG pathway genes occurs in persistent isolates from other CF patient samples. This question is particularly challenging to answer given our limited understanding of the metadata surrounding patient samples and the limited availability of longitudinal isolate sampling over long periods of time. Thus, while our study is limited to a single patient living with CF, the data herein lay a foundation for future longitudinal studies of A. fumigatus isolates from patients with CF to more fully define key selective pressures and pathoadaptive mechanisms of A. fumigatus in this important patient population.

To this end, a primary rationale for this study was to begin to address if and when antifungal therapy is warranted in CF patients positive for A. fumigatus (23, 74). Although recent studies have observed positive effects with voriconazole therapy in ABPA patients, in patients with CF, antifungal treatment varies widely across patients and centers (75). Drug interactions often preclude prolonged use of both azoles and CF medications, and the indeterminate clinical importance of treatment of A. fumigatus in CF further complicates treatment decisions (76). Moreover, studies have isolated azole-resistant isolates from patients with CF, often following initial azole treatment (23). In this regard, although the HOG pathway has been shown to be involved in antifungal resistance, the voriconazole resistance phenotype in clade 2 is surprising given that the AF100 patient has not been treated with azoles (77). Previous studies in patients with CF have also identified azole-resistant A. fumigatus isolates from azole-naive patients (78). While the *pbs2^C2^* allele did not significantly change the broth microdilution MIC of clade 2 isolates, it does contribute to increased hyphal voriconazole resistance, as evidenced by increased fungal growth in the presence of MIC-level voriconazole (Fig. 4). The observation of azole resistance and tolerance without prior azole therapy has also been observed in Clavispora (*Candida*) lusitaniae isolates from patients with CF (79). Thus, *in vivo* adaptation to the CF lung environment *per se* may promote increased antifungal drug tolerance across fungi associated with the CF lung, warrants further mechanistic study, and further emphasizes the importance of identifying persistent fungal isolates in patients with CF. Potentially, the presence of long-term A. fumigatus persistence in the CF lung may provide one indicator for the deployment of antifungal therapies. Taken together, our results argue that further understanding of A. fumigatus pathoadaptive mechanisms in CF is crucial for helping answer long-standing clinical questions regarding A. fumigatus and cystic fibrosis as well as the potential involvement of the HOG pathway in fungal *in vivo* persistence.

## MATERIALS AND METHODS

### Strain isolation and medium.

All growth assays were performed using a modified Aspergillus minimal medium (AMM) as described previously by Cove (80). Briefly, 10 g d-glucose (1% [wt/vol]) was dissolved in 20 ml of 50× salt solution, 1 ml trace elements solution, 66.666 ml 0.3 M l-glutamine solution (as nitrogen source, 20 mM final), and double-distilled water (ddH_2_O) to 1 liter (pH adjusted to 6.5 using NaOH). All media were made using 1.5% (wt/vol) agar (Beckton-Dickinson, Franklin Lakes, NJ) and autoclaved for at least 20 min at 121°C. Twenty milliliters were poured into plates after addition of filter-sterilized FeSO_4_ solution (70 mM, 2 mM final concentration). Standard growth conditions were 72 h at 37°C, 21% O_2_, and 5% CO_2_ in the dark, and all plate images were taken using a Canon Powershot SX40 HS camera, using automatic settings. Morphological characteristics were assessed as previously described (81). The liquid minimal medium used for mycelial DNA cultures consisted of 1% (wt/vol) d-glucose, 0.5% yeast extract ([wt/vol] Beckton-Dickinson), 20 ml 50× salt solution, 1 ml trace elements solution, and NaNO_3_ to 20 mM. Liquid medium was sterilized as described above.

All CF clinical isolates used in this study were cultured from expectorated sputum, collected from the AF100 patient during hospital visits (Fig. 1A). Sputum samples were collected and stored at −80°C by the Clinical Microbiology Laboratory at Dartmouth-Hitchcock Medical Center (DHMC) in collaboration with the Translational Research Core at Dartmouth. Isolates were obtained from the AF100 samples in one of two ways: (i) as individual colonies subcultured onto Sabouraud dextrose agar (Sab) slants following primary sputum culture on either Sab or blood agar by staff at the Clinical Microbiology Laboratory at DHMC, or (ii) as a collection of multiple colonies picked from sputum cultured on Sab plates for 30 h in the Cramer laboratory. All isolates were grown in pure culture for 3 days at 37°C, followed by single-spore isolation, propagation, and storage in −80°C glycerol stocks (25%). Single spores were collected by cutting individual germinated spores after 16 h of growth on AMM plates at 30°C and transferred to individual plates for further culture. All AF100 isolates were initially confirmed to be A. fumigatus using Sanger sequencing of PCR products from the internal transcribed spacer 1 (ITS1) region according to Schoch et al. (82), following spore DNA extraction as described by Fraczek et al. (83).

### Genome sequencing and assembly.

Genomic DNA (gDNA) was extracted from 24-h mycelia grown in station petri plates using the liquid medium described above, according to previously published methods (84). DNA concentration was quantified using a Qubit 2.0 fluorometer (Invitrogen) and the manufacturer’s recommended “broad range” protocol. Genomic sequencing was carried out on either Illumina NovaSeq 6000 or NextSeq 500 machines (see Table S1 in the supplemental material). DNA sequencing libraries were prepared using either the NEBNext Ultra II DNA library prep kit (for NovaSeq 6000 sequenced genomes) or the SeqOnce DNA library kit utilizing Covaris mechanized shearing (for the NextSeq 500 sequenced isolates), both according to the manufacturers’ recommendations with paired end library construction and barcoding for multiplexing.

### Phylogeny of strains.

Aligned SNPs from all isolates (identified as described below) were used to construct a phylogeny employing the maximum likelihood algorithm IQ-TREE (85). The best fit model for evolutionary rates was determined to be GTR+F+ASC according to the BIC score assessed with the ModelFinder function in IQ-TREE. The phylogeny was inferred under this model and 1,000 rapid bootstrap iterations. Individual branch support values were assessed using a Shimodaira-Hasegawa approximate likelihood ratio test (SH-aLRT) with 1,000 iterations. Tree visualization and trait mapping were carried out using the program Iroki (86).

### Phenotypic assays using CF-relevant stresses.

Phenotypic profiling assays were performed with cultures growing on AMM medium supplemented as indicated with treatments that induce CF-relevant stresses. The conditions tested included ambient oxygen (normoxia; 21% O_2_, 5% CO_2,_ 74% N_2_), low oxygen (hypoxic; 1% O_2_, 5% CO_2_, 94% N_2_), osmotic (2 M sorbitol), and oxidative (25 μM menadione) and azole antifungal drug (0.2 μg/ml voriconazole) stresses (59, 87). For conidium-based assays, conidia were collected from 3-day-old cultures, counted using a hemocytometer, and diluted to 500 spores/μl using 0.01% Tween 80. Two-microliter drops were inoculated onto plates and grown for 72 h under the above-indicated conditions. For hypha-based assays, conidia were plated as above and grown for 48 h under normoxia, except for TP-12.7, which was grown under hypoxia. A sterile 0.5-cm cork borer was used to transfer hyphal plugs from the leading, nonsporulating edge of the colonies to new plates with or without the above-indicated stresses, and plates were grown for 72 h under normoxia or hypoxia. Colony diameter was measured by averaging 2 perpendicular diameter measurements per plate. To compare the susceptibility of the isolates to a given stress, we quantified the ratio of the colony diameter under the stress condition to the colony diameter under the nonstressed growth conditions (H:N for hypoxia/normoxia or T:U for treated/untreated). Assays were performed in biological triplicates, and statistical analysis was performed in PRISM using one-way analysis of variance (ANOVA) with a multiple-comparisons posttest.

### Comparative genomics.

To examine the genetic diversity of the AF100 isolates in the context of global A. fumigatus diversity, raw sequence reads from 68 strains of A. fumigatus were downloaded from the NCBI database. These strains were combined with the 53 strains sequenced as part of this project, including 29 AF100 isolates (total number of strains analyzed, 121) (Table S1). The sequence reads for each strain were aligned to the AF293 reference genome downloaded from FungiDB v.46 (39, 88) using BWA v0.7.17 (89) and converted to the BAM file format using SAMtools v1.10 (90). Duplicate reads were marked for ignoring, and the BAM files were indexed using Picard tools v2.18.3 (http://broadinstitute.github.io/picard). To avoid overcalling variants near alignment gaps, reads were realigned using RealignerTargetCreator and IndelRealigner in the Genome Analysis Toolkit GATK v3.7 (91). Variants (SNPs and indels) were genotyped relative to AF293 using HaplotypeCaller in GATK v4.0 (92). Filtering was accomplished using GATK’s SelectVariants with the following parameters: for SNPs, -window-size = 10, -QualByDept < 2.0, -MapQual < 40.0, -QScore < 100, -MapQualityRankSum < −12.5, -StrandOddsRatio > 3.0, -FisherStrandBias > 60.0, and -ReadPosRankSum < −8.0; for indels, -window-size = 10, -QualByDepth< 2.0, -MapQualityRankSum < −12.5, -StrandOddsRatio > 4.0, -FisherStrandBias > 200.0, -ReadPosRank < −20.0, and -InbreedingCoeff < −0.8. Resultant variants were annotated with snpEff (93) relative to AF293. Variants that overlapped transposable elements (TEs) were removed by positional mapping to locations of annotated TEs in the FungiDB v.46 release of AF293 using BEDtools -subtract (94). For all analyses except the mutational accumulation analysis, variants annotated in snpEff as “intergenic,” “intron_variant,” and “synonymous_variant” were excluded from the analysis. Mutational accumulation analysis necessarily included variants annotated as “synonymous_variant.”

Variants were analyzed using a custom script implemented in the R programing environment (95) and assessed in three ways (95). First, to identify variants relevant to the tested phenotypes observed in the clade isolates, we targeted clade variants relative to four strains that failed to demonstrate these phenotypes under culture assay: the transient clinical strains TP-2, TP-7, and TP-11.1, isolated as part of this study, along with the nonclinical strain IF1SW-F4 previously isolated from the International Space Station, here referred to as the “out group” isolates (37). Second, to identify variants exclusive to each clade, we targeted variants in clade isolates relative to all nonclade isolates in the data set (*n* = 109). Finally, to look for recent signatures of adaptation to the human lung environment, variants in the clade 1 isolates were assessed relative to the putatively ancestral isolate TP-1.3, which was isolated at the first time point and fell directly outside clade 1 in the phylogenomic analysis.

### Positive selection analysis.

Using the outgroup targeted variant set, mutation accumulation curves were constructed for each clade using a custom R script (AF100_variant_analysis.R) that analyzed only those variants that had gone to fixation (present in a given time point, and all subsequent time points for that clade). Significance was assessed using linear regression at a *P* value of <0.05 in the R programing environment.

### Construct design, generation, and transformation for allelic exchange.

To generate the *pbs2* allele swap mutants, we designed primers (Table S2) and used fusion PCR to generate a construct using the *pbs2* genomic sequence from either TP-12.7 (*pbs2^C1^*) or TP-9 (*pbs2^C2^*) (Fig. 4A). We used the Prime Star high-fidelity *Taq* polymerase and buffer (TaKaRa Bio USA, Mountain View, CA) with 50 ng of template DNA and the following thermocycler settings: 95°C for 3 min followed by 35 cycles of 95°C for 10 s, 55°C for 15 s, and 72°C for 1 min/kb of amplicon, finishing with 72°C for 10 min. Constructs were confirmed via gel electrophoresis and extracted using the GeneJET gel extraction kit (Thermo Fisher Scientific, Waltham, MA). We used these constructs for transformation via homologous recombination in TP-9 protoplasts generated with lysing enzymes from *Trichoderma* spp. (Sigma-Aldrich, St. Louis, MO, USA). Transformation was performed according to previously published protocols (81).

10.1128/mBio.02153-21.7 TABLE S2 Primer sequences used in this study. Download Table S2, DOCX file, 0.01 MB. Copyright © 2021 Ross et al. 2021 Ross et al. https://creativecommons.org/licenses/by/4.0/ This content is distributed under the terms of the Creative Commons Attribution 4.0 International license.

### Protein extraction and Western blotting.

Mycelia from four 24-h stationary liquid AMM cultures in 12-well plates (1 × 10^6^ spores/ml) were pooled and transferred to either fresh AMM or AMM with 1 M NaCl for 15 min and then flash frozen, lyophilized overnight, and bead beaten for 1 min with 2.3-mm beads. Protein was extracted using the extraction buffer detailed by Bruder Nascimento et al. (96) (per 10 ml: 10% [vol/vol] glycerol, 0.1% [vol/vol] SDS, 1% [vol/vol] Triton X-100, 50 mM Tris-HCl [pH 7.5], 150 mM NaCl, 5 mM EDTA, 15 mM EGTA, 100 μl HALT protease inhibitor cocktail [Thermo Scientific], 1 mM phenylmethylsulfonyl fluoride [PMSF], 50 mM sodium fluoride, 5 mM sodium pyrophosphate, 5 mM sodium orthovanadate, 50 mM β-glycerophosphate, ddH_2_O to 10 ml). One milliliter of buffer was added to powdered mycelia and vortexed, and tubes were centrifuged at 13,000 rpm for 5 min. Approximately 500 μl was removed, and protein was quantified via Bradford analysis. Forty micrograms of protein was used for each sample for Western blot analysis. Proteins were transferred from a 10% SDS-PAGE gel onto a nitrocellulose membrane for a Western blot assay using the Trans-Blot turbo transfer system (Bio-Rad, Hercules CA). Phosphorylated SakA (P-SakA) was detected using a rabbit anti-P-p38 antibody (number 9215; Cell Signaling Technology, Danvers, MA) at 1:1,000 dilution. SakA-FLAG was detected using the monoclonal anti-FLAG M2 antibody (F3165; Sigma-Aldrich) at 1:2,000 dilution. Fluorescence imaging and quantification were performed using the LI-COR Odyssey CLx system and the manufacturer’s pan/phospho protocol, along with REVERT total protein stain for normalization (LI-COR Biosciences, Lincoln, NE).

### RNA extraction and qRT-PCR.

Mycelia from 30-h stationary liquid AMM cultures (1 × 10^6^ spores/ml) were used for medium transfer and RNA extraction. Mycelia were transferred to either fresh AMM or AMM with 1 M NaCl for 15 min and then flash frozen, lyophilized overnight, and bead beaten for 1 min with 2.3-mm beads. Homogenate was suspended in 1 ml of TRIsure (Meridian Biosciences, Cincinnati, OH), and RNA was extracted as previously described (97). For quantitative reverse transcription-PCR (qRT-PCR), 5 μg of RNA was treated with the Ambion Turbo DNase kit (Thermo Fisher Scientific, Waltham, MA) according to the manufacturer’s protocol, and DNase-treated RNA was reverse transcribed as previously described (97). Statistical analysis was performed with one-way ANOVA with a Dunnett posttest for multiple comparisons. Data were collected on a CFX Connect real-time PCR detection system (Bio-Rad, Hercules CA) with CFX Maestro software.

### Data availability.

All genome data generated as part of this project was deposited into the NCBI Short Read Archive. SRA accession numbers can be found in Table S3 in the supplemental material. The code and data files for variant assessment associated with this project can be found on GitHub in the repository at https://github.com/stajichlab/AF100 or https://zenodo.org/record/4409346#.YRGGiPKSmF4.

10.1128/mBio.02153-21.9 TABLE S3 NCBI Short-Read Archive accession numbers for all sequenced isolates in this study. Download Table S3, DOCX file, 0.02 MB. Copyright © 2021 Ross et al. 2021 Ross et al. https://creativecommons.org/licenses/by/4.0/ This content is distributed under the terms of the Creative Commons Attribution 4.0 International license.
