# Supplementary material for: Aspergillus fumigatus In-Host HOG Pathway Mutation for Cystic Fibrosis Lung Microenvironment Persistence
Source: mBio. 2021 Aug 31;12(4):e02153-21. doi: 10.1128/mBio.02153-21 (PMC8406193; doi:10.1128/mBio.02153-21)
Supplement: TABLE S1 [file mbio.02153-21-st001.docx]

| Isolate | Name for NCBI Submission | Depth | N Variants | N Nonsynonymous Variants | N Missense Variants |
| --- | --- | --- | --- | --- | --- |
| TP-10.5 | AF100-10_5 | 24 | 31839 | 22729 | 9732 |
| TP-10.1 | AF100-10B | 91 | 27864 | 20048 | 8426 |
| TP-11.3 | AF100-11_3 | 40 | 31776 | 22681 | 9724 |
| TP-11a | AF100-11A | 39 | 28599 | 20447 | 8663 |
| TP-11.1 | AF100-11B | 22 | 28591 | 20437 | 8669 |
| TP-12e | AF100-12_10 | 35 | 27769 | 19721 | 8403 |
| TP-12a | AF100-12_2 | 33 | 26789 | 19126 | 8185 |
| TP-12f | AF100-12_21 | 40 | 29349 | 20982 | 8959 |
| TP-12g | AF100-12_24 | 36 | 29253 | 20734 | 9102 |
| TP-12h | AF100-12_37 | 29 | 29168 | 20869 | 8899 |
| TP-12b | AF100-12_3G | 31 | 27452 | 19526 | 8499 |
| TP-12c | AF100-12_5 | 68 | 25952 | 18367 | 8063 |
| TP-12.7 | AF100-12_7 | 32 | 27776 | 19973 | 8407 |
| TP-12d | AF100-12_7G | 30 | 22579 | 15727 | 7104 |
| TP-1b | DMC2_AF100-1_14 | 83 | 26679 | 19059 | 8123 |
| TP-1e | DMC2_AF100-1_20_C | 70 | 28846 | 20603 | 8730 |
| TP-1d | DMC2_AF100-1_24 | 96 | 28219 | 20164 | 8683 |
| TP-1.3 | DMC2_AF100-1_3 | 146 | 26196 | 18538 | 8070 |
| TP-1a | DMC2_AF100-1_8 | 39 | 27414 | 19311 | 8479 |
| TP-12.9 | DMC2_AF100-12_9 | 52 | 32015 | 22845 | 9818 |
| TP-2 | DMC2_AF100-2B | 51 | 27116 | 19130 | 8332 |
| TP-3 | DMC2_AF100-3B | 55 | 27773 | 19963 | 8400 |
| TP-4 | DMC2_AF100-4B | 46 | 27806 | 19989 | 8414 |
| TP-5 | DMC2_AF100-5B | 56 | 27809 | 19995 | 8414 |
| TP-6 | DMC2_AF100-6B | 75 | 27797 | 19980 | 8415 |
| TP-7 | DMC2_AF100-7B | 63 | 30260 | 21602 | 9296 |
| TP-8 | DMC2_AF100-8B | 67 | 31561 | 22496 | 9675 |
| TP-9 | DMC2_AF100-9B | 82 | 31866 | 22726 | 9781 |
| TP-1c | DMC2_AF1001_15 | 70 | 27106 | 19115 | 8327 |
