# Supplementary figures and images for: Aspergillus fumigatus In-Host HOG Pathway Mutation for Cystic Fibrosis Lung Microenvironment Persistence
Source: mBio. 2021 Aug 31;12(4):e02153-21. doi: 10.1128/mBio.02153-21 (PMC8406193; doi:10.1128/mBio.02153-21)

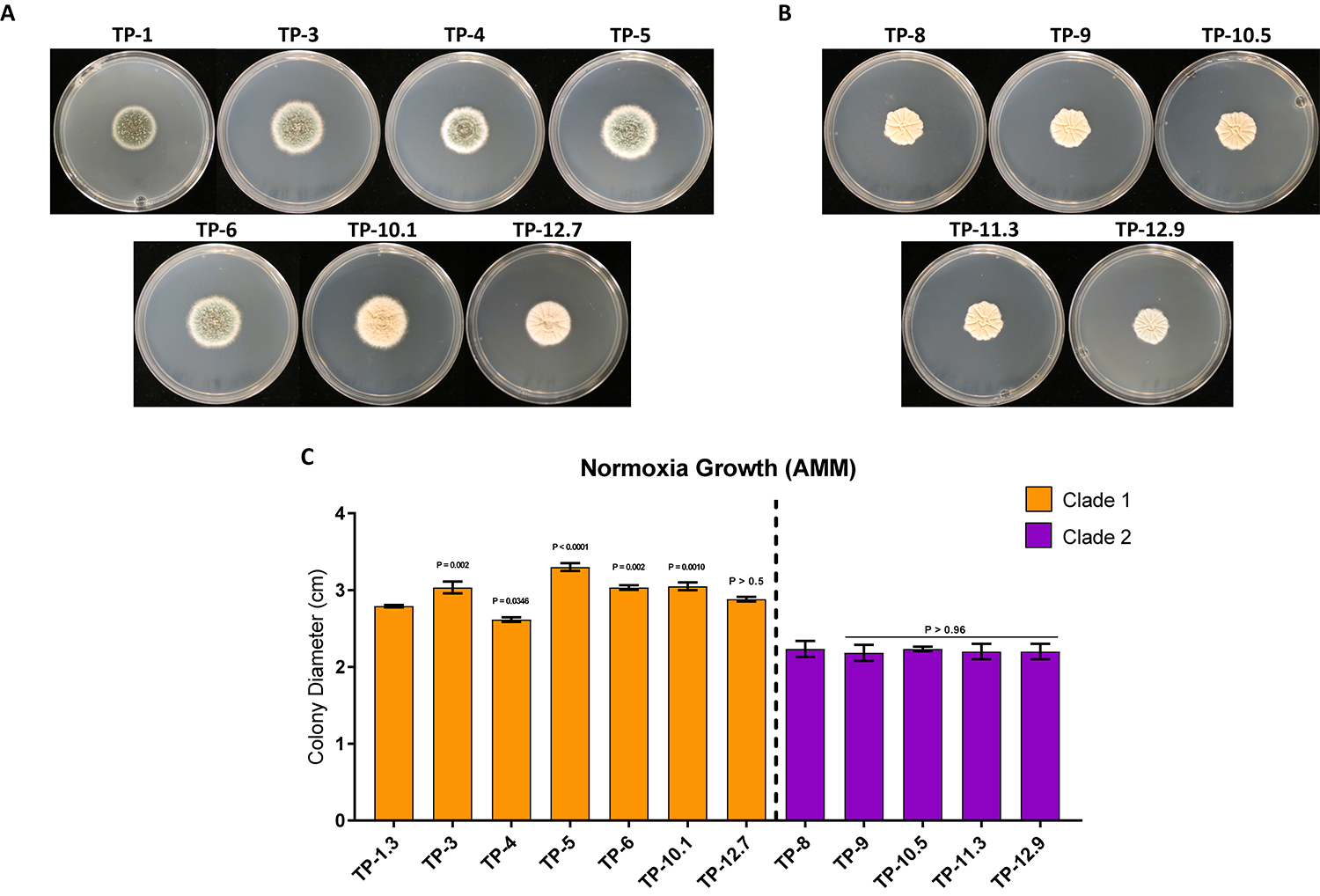

Supplement: FIG S1 [file mbio.02153-21-sf001.tif]

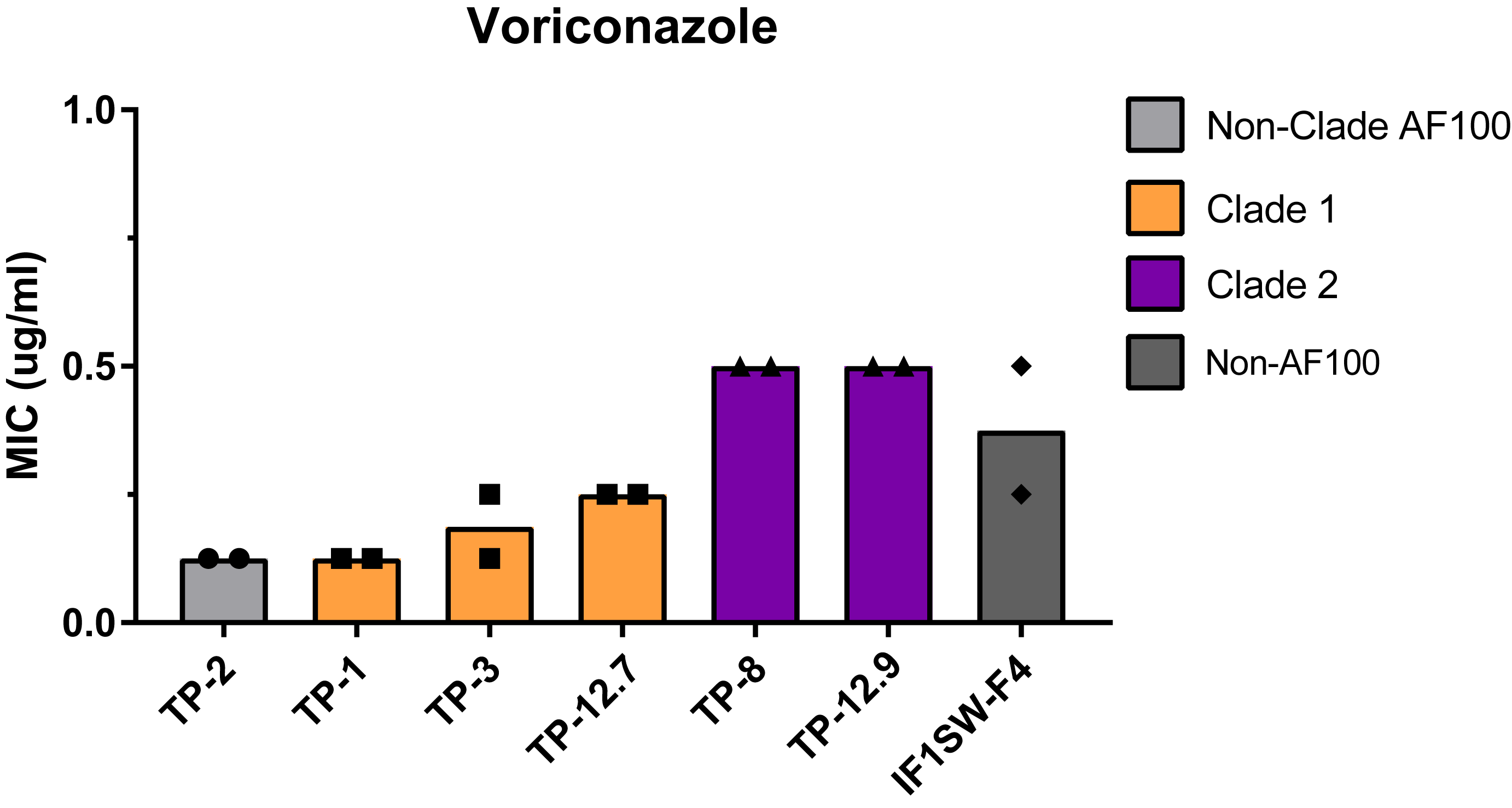

Supplement: FIG S2 [file mbio.02153-21-sf002.tif]

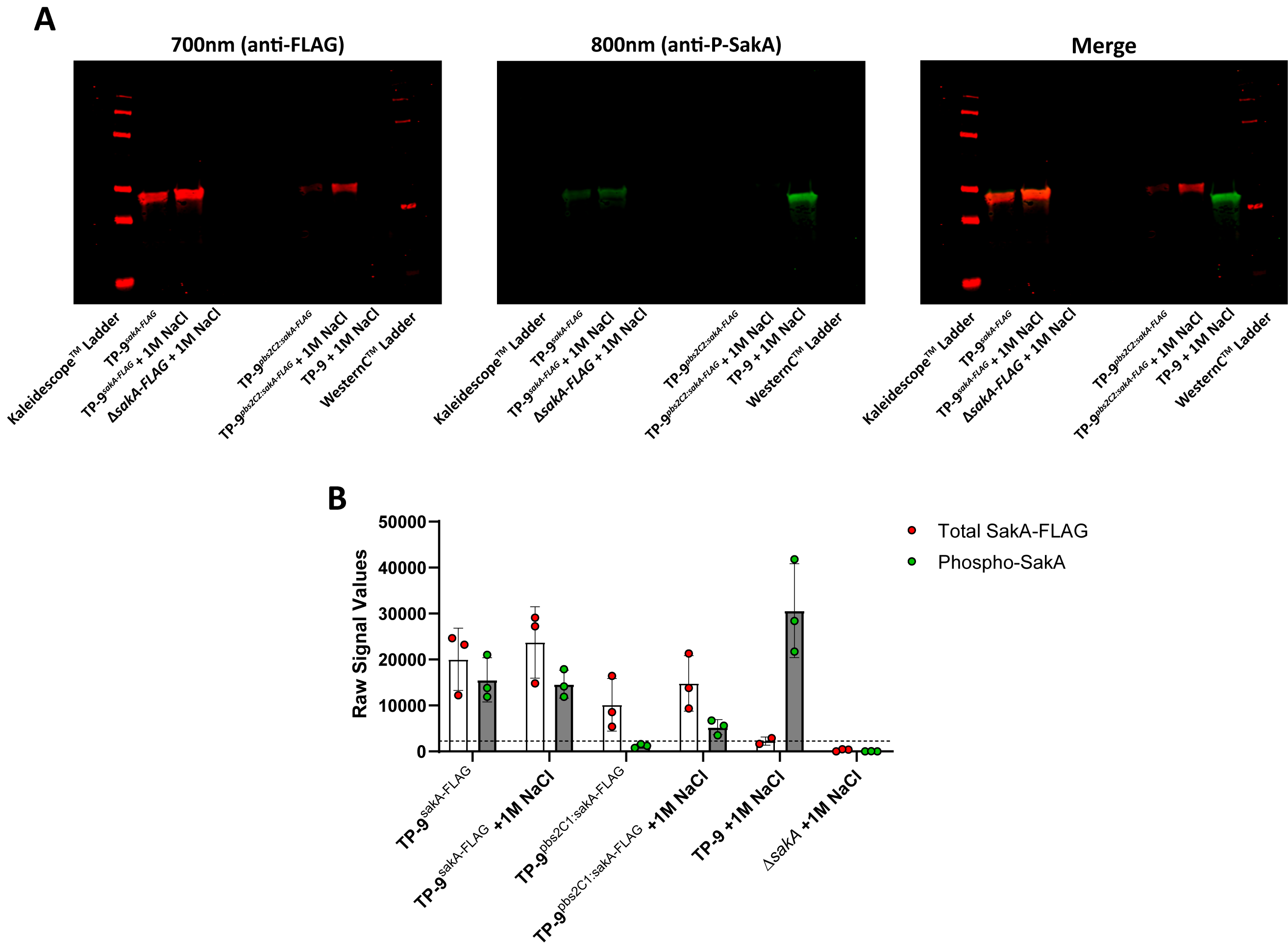

Supplement: FIG S4 [file mbio.02153-21-sf004.tif]

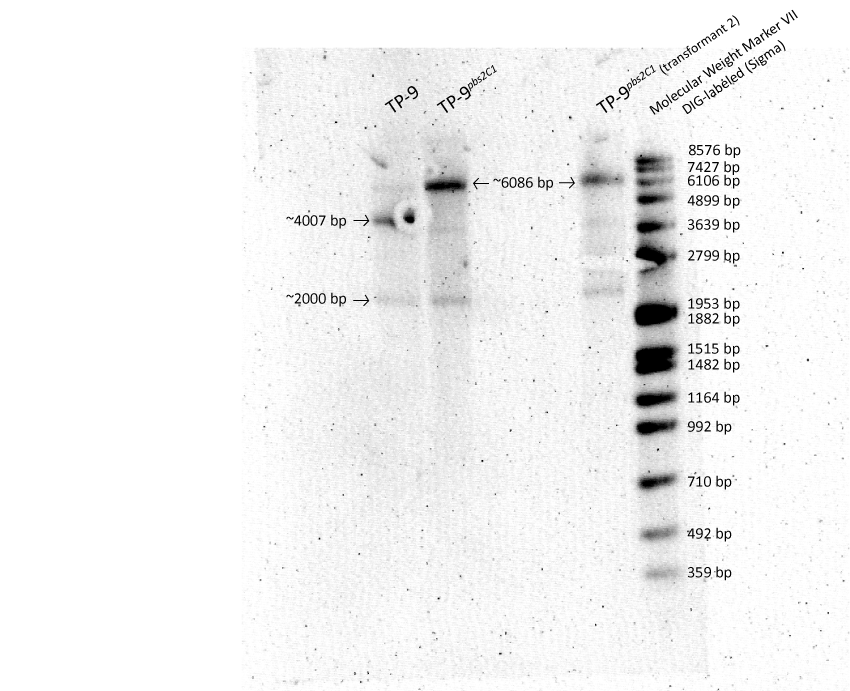

Supplement: FIG S5 [file mbio.02153-21-sf005.tif]

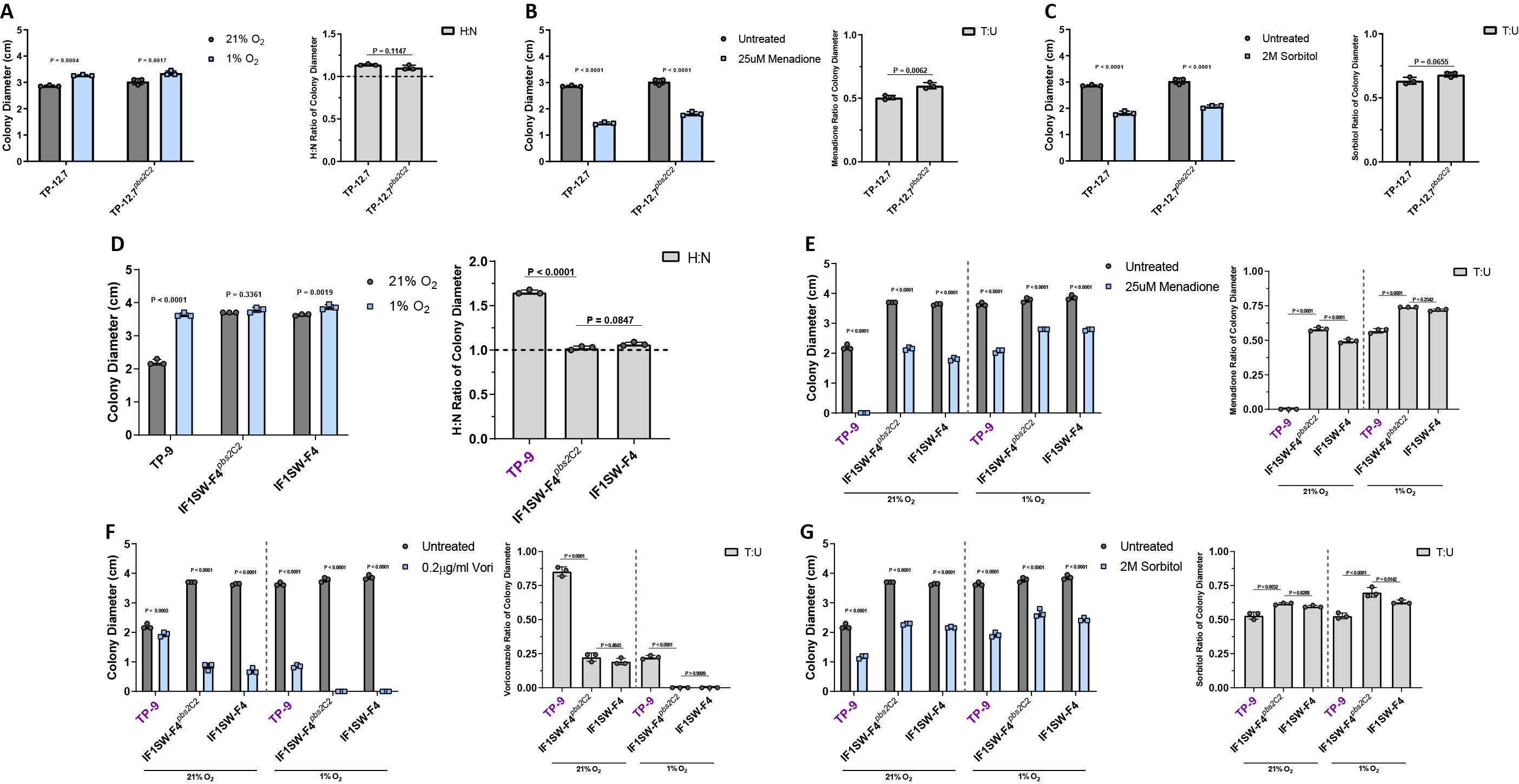

Supplement: FIG S3 [file mbio.02153-21-sf003.tif]
