# Supplementary material for: Aspergillus fumigatus In-Host HOG Pathway Mutation for Cystic Fibrosis Lung Microenvironment Persistence
Source: mBio. 2021 Aug 31;12(4):e02153-21. doi: 10.1128/mBio.02153-21 (PMC8406193; doi:10.1128/mBio.02153-21)
Supplement: TABLE S2 [file mbio.02153-21-st002.docx]

| Primer Name | Sequence (5'-3') | Description |
| --- | --- | --- |
| RAC 4632 | gccaacgtaccaacgagagaaaac | F primer for 5' flank used to generate *pbs2* allelic exchange construct |
| RAC 4633 | ctgtgccgccagtcaccaccggtcgcctcaaacaatgctct | R primer for 5' flank used to generate *pbs2* allelic exchange construct |
| RAC 4634 | cgcatcagtgcctcctctcagaccaaatctccaaccccgaacgc | F primer for 3' flank used to generate *pbs2* allelic exchange construct |
| RAC 4635 | caaggaaggagcgatgggaca | R primer for 3' flank used to generate *pbs2* allelic exchange construct |
| RAC 2055 | accggtcgcctcaaacaatgctct | F primer to amplify *ptrA* marker from |
| RAC 2056 | cgcatcagtgcctcctctcagac | R primer to amplify *ptrA marker* for allelic exchange construct |
| RAC 4636 | ctcgaccacccacagcc | F primer for fusion PCR |
| RAC 4637 | cacaactgcccatgattcggc | R primer for fusion PCR |
| RAC 4612 | cctcatgctgtctctaccgga | F primer for Sanger sequencing of *pbs2* allele |
| RAC 31 | tccgtaggtgaacctgcgg | F primer for the Internal Transcriber Spacer, used for species ID |
| RAC 32 | tcctccgcttattgatatgc | R primer for the Internal Transcriber Spacer, used for species ID |
| RAC 158 | tcactgcccttgctccctcgtc | F qRT-PCR primer for the *actA* housekeeping gene |
| RAC 159 | gcacttgcggtgaacgatcgaa | R qRT-PCR primer for the *actA* housekeeping gene |
| RAC 7 | ataatgttcagaccgccctctgct | F qRT-PCR primer for the *tubA* housekeeping gene |
| RAC 8 | gacggatgtggaattgcccacaaa | R qRT-PCR primer for the *tubA* housekeeping gene |
| RAC 3415 | cctgttgcttgctcacaa | F qRT-PCR primer for *atfA* |
| RAC 3416 | taccctgagtagggataggt | R qRT-PCR primer for *atfA* |
| RAC 6133 | gctcagactctccatcgtcctct | F qRT-PCR primer for *yap1* |
| RAC 6134 | aggcgactgagagtagcactcc | R qRT-PCR primer for *yap1* |
| RAC 6135 | taaccgccacgtcatcaaca | F qRT-PCR primer for *gpd1* |
| RAC 6136 | cccgtacttccatgtgcaggt | R qRT-PCR primer for *gpd1* |
| RAC 165 | atacggaagagttagccggtgctt | F qRT-PCR primer for *tpsA* |
| RAC 166 | tctgttcagctcggtgacaaagga | R qRT-PCR primer for *tpsA* |
