# Supplementary material for: Aspergillus fumigatus In-Host HOG Pathway Mutation for Cystic Fibrosis Lung Microenvironment Persistence
Source: mBio. 2021 Aug 31;12(4):e02153-21. doi: 10.1128/mBio.02153-21 (PMC8406193; doi:10.1128/mBio.02153-21)
Supplement: TABLE S3 [file mbio.02153-21-st003.docx]

| NCBI Short-Read Archive Accession | Study ID | Sample ID |
| --- | --- | --- |
| SRR12763076 | TP-12h | A_fum_AF100-12_37 |
| SRR12763077 | TP-12g | A_fum_AF100-12_24 |
| SRR12763078 | TP-12f | A_fum_AF100-12_21 |
| SRR12763079 | TP-12a | A_fum_AF100-12_2 |
| SRR12763080 | 204NB-8 | A_fum_204NB-8 |
| SRR12763081 | 84-NIH | A_fum_84_NIH |
| SRR12763082 | Oryx-1 | A_fum_Oryx_1 |
| SRR12763083 | TP-12e | A_fum_AF100-12_10 |
| SRR12763084 | Moose_3­_1_2 | A_fum_Moose_3_1_2 |
| SRR12763085 | Mg18-1 | A_fum_Mg18-1 |
| SRR12763086 | TP-1c | A_fum_AF1001_15 |
| SRR12763087 | TP-9 | A_fum_AF100-9B |
| SRR12763088 | TP-8 | A_fum_AF100-8B |
| SRR12763089 | TP-7 | A_fum_AF100-7B |
| SRR12763090 | TP-6 | A_fum_AF100-6B |
| SRR12763091 | TP-5 | A_fum_AF100-5B |
| SRR12763092 | TP-4 | A_fum_AF100-4B |
| SRR12763093 | TP-3 | A_fum_AF100-3B |
| SRR12763094 | TP-11.1 | A_fum_AF100-11B |
| SRR12763095 | TP-2 | A_fum_AF100-2B |
| SRR12763096 | TP-12.9 | A_fum_AF100-12_9 |
| SRR12763097 | TP-1a | A_fum_AF100-1_8 |
| SRR12763098 | TP-1.3 | A_fum_AF100-1_3 |
| SRR12763099 | TP-1d | A_fum_AF100-1_24 |
| SRR12763100 | TP-1e | A_fum_AF100-1_20_C |
| SRR12763101 | TP-1b | A_fum_AF100-1_14 |
| SRR12763102 | B8783_CDC-17 | A_fum_B8783_CDC-17 |
| SRR12763103 | B5960_CDC-29 | A_fum_B5960CDC_29 |
| SRR12763104 | B5461_CDC-2 | A_fum_B5461_CDC-2 |
| SRR12763105 | TP-11a | A_fum_AF100-11A |
| SRR12763106 | B5460_CDC-13 | A_fum_B5460_CDC-13 |
| SRR12763107 | B5289_CDC-25 | A_fum_B5289_CDC-25 |
| SRR12763108 | B5288_CDC-16 | A_fum_B5288_CDC-16 |
| SRR12763109 | B5269_CDC-24 | A_fum_B5269_CDC-24 |
| SRR12763110 | B5259_CDC-23 | A_fum_B5259_CDC-23 |
| SRR12763111 | B5258_CDC-22 | A_fum_B5258CDC_22 |
| SRR12763112 | B5224_CDC-21 | A_fum_B5224_CDC-21 |
| SRR12763113 | B1821_CDC-8 | A_fum_B1821_CDC-8 |
| SRR12763114 | B10540_CDC-20 | A_fum_B10540_CDC-20 |
| SRR12763115 | AFUG_100413-0667 | A_fum_AFUG_100413-0667 |
| SRR12763116 | TP-11.3 | A_fum_AF100-11_3 |
| SRR12763117 | AFUG_082015-1200 | A_fum_AFUG_082015-1200 |
| SRR12763118 | AFUG_031815-1869 | A_fum_AFUG_031815-1869 |
| SRR12763119 | AFIS_1435_CDC-6 | A_fum_AFIS1435CDC_6 |
| SRR12763120 | AFIS_23107_CDC-12 | A_fum_AFIS_23107_CDC-12 |
| SRR12763121 | AFIS_2302_CDC-18 | A_fum_AFIS_2302_CDC-18 |
| SRR12763122 | AFIS_13708_CDC-14 | A_fum_AFIS_13708_CDC-14 |
| SRR12763123 | TP-12d | A_fum_AF100-12_7G |
| SRR12763124 | TP-12.7 | A_fum_AF100-12_7 |
| SRR12763125 | TP-12c | A_fum_AF100-12_5 |
| SRR12763126 | TP-12b | A_fum_AF100-12_3G |
| SRR12763127 | TP-10.1 | A_fum_AF100-10B |
| SRR12763128 | TP-10.5 | A_fum_AF100-10_5 |
